# Tumor Microenvironment‐Triggered Aggregated Magnetic Nanoparticles for Reinforced Image‐Guided Immunogenic Chemotherapy

**DOI:** 10.1002/advs.201802134

**Published:** 2019-01-29

**Authors:** Qinjun Chen, Lisha Liu, Yifei Lu, Xinli Chen, Yujie Zhang, Wenxi Zhou, Qin Guo, Chao Li, Yiwen Zhang, Yu Zhang, Donghui Liang, Tao Sun, Chen Jiang

**Affiliations:** ^1^ Key Laboratory of Smart Drug Delivery Ministry of Education State Key Laboratory of Medical Neurobiology Department of Pharmaceutics School of Pharmacy Fudan University Shanghai 201203 China

**Keywords:** antitumor immune responses, immunogenic cell death, magnetic nanoparticles, reactive oxygen species, triggered release

## Abstract

Anticancer therapies, which can induce cell death and elevate antitumor immune response in the meantime, are considered as effective treatments for many types of cancers. Immunogenic cell death (ICD) induced by chemodrugs is a promising and typical strategy to achieve cell cytotoxicity and immunological enhancement together. However, due to the low level of ICD induction and less tumor‐targeting accumulation, application of traditional ICD inducers is limited. Here, tumor‐targeting core–shell magnetic nanoparticles (ETP‐PtFeNP:α‐enolase targeting peptide modified Pt‐prodrug loaded Fe_3_O_4_ nanoparticles) are developed to reinforce ICD induction of loaded‐oxaliplatin (IV) prodrug. After tumor‐targeting accumulation and endocytosis, platinum (IV) complexes are activated by intracellular reductive elimination to yield and release the Pt (II) congener, oxaliplatin, leading to DNA lesions and reactive oxygen species (ROS) generation. Simultaneously, in‐progress‐released ferric ions elicit highly toxic ROS (·OH or ·OOH) burst and interfere with the intracytoplasmic redox balance (like endoplasmic reticulum stress), leading to ICD‐associated immunogenicity enhancement and specific antitumor immune responses to kill the tumor cells synergistically. Meanwhile, the transverse relaxation rate *R*_2_ of ETP‐PtFeNP is remarkably increased by more than three times while triggered by reductant, suggesting ETP‐PtFeNP a high‐sensitivity *T*_2_ contrast agent for magnetic resonance imaging.

During recent years, companied with the rise of immunotherapy, anticancer therapies that can stimulate cancer cells to undergo an immunogenic apoptosis and induce an effective antitumor immune response in tumor tissues have attracted much attention in the research communities of oncology, termed as immunogenic cell death (ICD).^1^ The immunogenic characteristics of ICD are mainly mediated by the exposure of damage associated molecular patterns (DAMPs), which includes surface exposure of calreticulin (CRT), ATP secretion, and high mobility group protein B1 (HMGB1) release.^2^ Particularly, the surface‐exposed CRT serves as an engulfment signal that targets apoptotic cells to dendritic cells (DCs), subsequently, leads to cross‐presentation of tumor antigens and antitumor specific T‐cell responses.^3^ Meanwhile, at the postapoptotic stage, the HMGB1 is released into the extracellular milieu to facilitate the immunogenicity of CRT by interacting with several receptors expressed on the surface of DCs (such as toll‐like receptor 4).^4^

Mechanistically, there are several studies indicating that the ICD induction requires rapid reactive oxygen species (ROS) generation and further ROS‐based endoplasmic reticulum (ER) stress, both of which synergistically activate danger signaling pathways that contributes to the extracellular DAMPs mobilization.^5^ Accordingly, the ICD inducers are classified as two major types: type I, modalities that induce cell death through non‐ER associated targets but stimulate ICD‐associated danger signaling through collateral ER stress effects (most of ICD inducers belong to this category, e.g., oxaliplatin, anthracyclines, mitoxantrone, and cyclophosphamide); type II, modalities that selectively target the ER to induce cell death and ICD‐associated immunogenicity in an ER‐focused manner (only a few therapies induce ICD via this way, e.g., hypericin‐based photodynamic therapy). However, it has recently been demonstrated that the ICD‐associated immunogenicity fostered by type I inducers was not as favorable as expected, but that induced by focused ER stress was quite effective.^6^ In view of the pervasive application of type I ICD inducers in clinic,[[qv: 2b]] it is promising and necessary to develop an alternative treatment strategy to enhance the ICD‐associated immunogenicity of type I ICD inducers and further elicit potent antitumor immune responses.

Platinum‐based drugs are the cornerstones of chemotherapeutics, and have been used in 80% of clinical therapeutic regimens. The third‐generation star product, oxaliplatin, not only shows a potent effect in chemotherapy, but also has a fine capability in ICD induction.^7^ Furthermore, recent advances have revealed that the interaction between PD‐L2 and PD‐1 also plays a pivotal role in PD‐1 mediated immunosuppression, and oxaliplatin could down‐regulate the PD‐L2 expression to reactivate the T cell cytotoxic capacity on the immunosuppressive microenvironment, via the inhibition of STAT6‐mediated expression pathways.^8^

Since discovered in 2012, ferroptosis has been widely known as an iron‐ and ROS‐dependent form of cell death.^9^ Catalyzed by Fe^3+^ or Fe^2+^ (so‐called Fenton's reaction), the intracellular hydrogen peroxide (H_2_O_2_) would be rapidly converted to highly reactive hydroxyl radical (·OH), causing a high ROS stress and resulting in irreversible oxidative damage to lipids, proteins and DNA, eventually cell ferroptosis.^10^ More importantly, several studies have found that ferroptotic agents could induce an unfolded protein response and subsequently activated a series of ER stress‐mediated ferroptotic signaling pathways,^11^ which fits in with our demand for enhanced treatment strategy. As typical and FDA‐approved iron‐based nanomaterial, iron oxide nanoparticles have been widely employed in cancer diagnosis, drug delivery, and ferroptosis induction. It can be degraded by acidic compounds or reductant, and then the metabolized ferric/ferrous ions actively participate in intracellular Fenton's reaction, causing a burst of ROS.^9^ In addition, nanocarrier‐drug delivery systems have been extensively explored for cancer drug delivery to prolong the blood circulation and increase tumor accumulation, through enhanced permeation and retention (EPR) effect or active tumor‐targeting.^12^ Therefore, employment of the iron oxide nanoparticle as a multifunctional vector to deliver ICD inducers may can achieve the goal of reinforced immunogenic chemotherapy.

Herein, we reported on a tumor‐targeting core–shell magnetic nanoparticle (ETP‐PtFeNP) to reinforce the ICD induction of loaded‐oxaliplatin (IV) prodrug. As designed, the formulation consists of two parts, Fe_3_O_4_ core and drug‐loaded polymeric shells (**Scheme** **1**). First, we explored the synthesis of polymeric shell as illustrated in Scheme S1 of the Supporting Information. Through a series of modular conjugation including esterification, amidation reaction, ring‐opening reaction (ROP), click reaction, we obtained the polymeric shells (tumor‐targeting polymer, ETP‐OXA‐DHAC or nontargeting polymer, PEG‐OXA‐DHAC). Meanwhile, we successfully characterized the intermediates and final compound by mass spectrometry, proton nuclear magnetic resonance spectra (^1^H‐NMR), infrared adsorption spectrum, high performance liquid chromatography, and gel permeation chromatography, which are shown in Figures S1–S18 of the Supporting Information. The oleic acid‐Fe_3_O_4_ nanoparticles were found of uniform size in tetrahydrofuran with modification of oleic acid (Figure S19, Supporting Information).

**Scheme 1:**
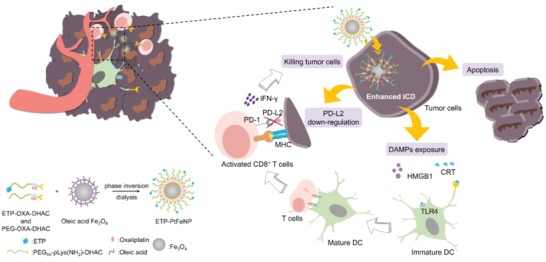
Representation of preparation and elevated antitumor immune responses with ETP‐PtFeNP treatment.

Subsequently, biocompatible prodrug‐loaded Fe_3_O_4_ nanoparticles, ETP‐PtFeNP and PtFeNP, were obtained via the competitive binding interaction between the terminal catechol group of polymers (ETP‐OXA‐DHAC/PEG‐OXA‐DHAC) and the carboxyl group of oleic acid (phase inversion dialysis method, Scheme 1). Compared with hydrophobic oleic acid‐Fe_3_O_4_, the freshly prepared ETP‐PtFeNP showed fine water dispersion stability (Figure S20, Supporting Information). Dynamic light scattering (DLS) and transmission electron microscopy (TEM) results showed that ETP‐PtFeNPs were spherical in shape with an averaged hydrodynamic diameter of 24 nm and morphological diameter of 7 nm (**Figure** **1**a), respectively. Besides, the DLS and TEM results of nontargeting nanoparticles, PtFeNP, were similar to ETP‐PtFeNP (Figure 1b,c), indicating that modification with targeting moiety did not significantly change the physicochemical property of the formulation, which is conducive to the subsequent targeting investigation. The formulations still maintained fine particle diameters after 7‐day incubation with PBS 7.4 (Figure 1d), and the surface charge of ETP‐PtFeNP and PtFeNP were ≈−10.9 and −11.3 mV (Figure 1c), respectively, which could be translated to high colloidal stability in water.^12^ In addition, the loading content of Pt and Fe in PtFeNP were 11.6 and 5.6 w/w% (ICP‐AES), respectively, suggesting a relatively high loading of oxaliplatin at 23.5 w/w%. Meanwhile, owing to α‐enolase targeting peptide (ETP) modification, ETP‐PtFeNP exhibited a little lower Pt and Fe loading than PtFeNP, with Pt and Fe loading of 10.7 w/w% and 5.2 w/w% (ICP‐AES), respectively.

**Figure 1:**
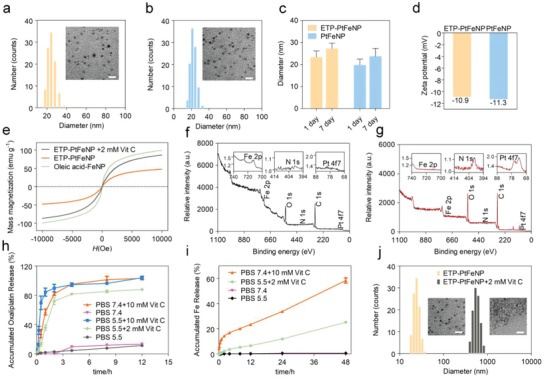
Characterization of ETP‐PtFeNP formulations. a,b) DLS profile and TEM image of ETP‐PtFeNP and PtFeNP, respectively. Scale bars: 10 nm for inset TEM image. c) The hydrous diameters of ETP‐PtFeNP and PtFeNP before and after stored in PBS 7.4 for 7 days. d) Zeta‐potential of ETP‐PtFeNP and PtFeNP after stored in PBS 7.4 for 7 days. e) The magnetization curves for different formulations. H, magnetic field. f,g) XPS deconvoluted spectra of oleic‐acid Fe_3_O_4_ and ETP‐PtFeNP, respectively. h) Oxaliplatin release profile of ETP‐PtFeNP nanoparticles under different conditions. Data are presented as means ± SD (*n* = 3). i) Fe release profile of ETP‐PtFeNP nanoparticles under different conditions. Data are presented as means ± SD (*n* = 3). j) DLS profiles and TEM images of ETP‐PtFeNP before and after incubation with 2 × 10^−3^ m Vit C for 3 h. Scale bars: 10 nm for inset TEM image.

To verify that whether the polymeric shell has been successfully coated to Fe_3_O_4_ core, we performed magnetization measurements, for that the saturation magnetization of magnetic materials would be changed dramatically if they were modified with polymers.^13^ As shown in Figure 1e, the remarkably declined saturation magnetization of nanoparticles, from 99.28 to 47.65 emu g^−1^, revealed the successful shielding of polymeric shell. Moreover, the X‐ray photoelectron spectroscopy (XPS) analysis was carried out to quantify the compositional and chemical states on the surface of nanoparticles.^14^ Compared with oleic acid‐Fe_3_O_4_, the declined Fe2p concentrations in ETP‐PtFeNP, from 4.22% to 0.08%, and the increased N1s and Pt4f7 concentrations in ETP‐PtFeNP, from 0.25% and 0.02% to 1.09% and 0.16%, respectively, both demonstrated the successful preparation of core–shell nanoparticles.

After the successful preparation of ETP‐PtFeNP nanoparticles, we evaluated the release of oxaliplatin (II) from the ETP‐PtFeNP formulation under several in vivo simulated environments. As shown in Figure 1h and Figure S22 (Supporting Information), in PBS 7.4, 10 × 10^−3^ m Vitamin C (Vit C) (to stimulate general intracellular reductive condition^15^) or PBS 5.5, 2 × 10^−3^ m Vit C (to stimulate the reductive condition in lysosomes^16^), nearly 80% of loaded oxaliplatin was released during the initial 4 h. By contrast, without the addition of Vit C, only 13% of oxaliplatin was found after the 12 h release (Figure S23, Supporting Information). Interestingly, obvious aggregation of nanoparticles occurred when most of oxaliplatin was released (Figure 1j). Based on the stereostructure study of polymeric shells,^17^ we suspected that the induced aggregation could be ascribed by the declined steric hindrance that was initiated by the oxaliplatin release. To address the hypothesis, we further prepared a series of nanoparticles that were modified with several synthetic intermediates (compound **8**, **10**), and investigated their water dispersion stability. However, all the prepared nanoparticles were unstable in water, as shown in Figure S24 of the Supporting Information, even for the one that contains PEG‐*p*Lys (cbz)‐DHAC, which had a similar molecular structure to PEG‐OXA‐DHAC. Thus, there may be some unconventional factors that affected the dispersion stability. It has been reported that the large spin–orbit (s–o) coupling between 5d element (Pt) and 3d element (Fe) could contribute to the decreased saturation magnetization of Fe–Pt alloys.^18^ Our findings could be explained well by this theory that when oxaliplatin existed inside the nanoparticles, via a shielding effect, nanoparticles could maintain stable structures, once oxaliplatin was released, shielding effect was impaired, resulting in aggregation of nanoparticles. Moreover, as shown in Figure 1e, the Vit C‐processed nanoparticles indeed had twofold increased saturation magnetization than unprocessed nanoparticles, ≈86.21 emu g^−1^, and still possessed a superparamagnetic property, which were thought to facilitate the enhancement of *T*_2_‐weight MRI effect.

Next, the investigation of ferric ions release was carried out. Nearly 25% of ferric ions were released from the formulations in PBS 5.5, 2 × 10^−3^ m Vit C after 48 h incubation, while in PBS 5.5 or PBS 7.4, only up to 0.6% of ferric ions were detected after 48 h incubation (Figure 1i). Due to the lower hydrogen ion concentrations in us in vivo stimulated conditions than other reported conditions,^19^ it was understandable that our results were differed from the common understanding of acidic degradation of Fe_3_O_4_ nanoparticles. Meanwhile, we found that in PBS 7.4, 10 × 10^−3^ m Vit C condition, twice over released ferric ions was detected at 48 h than in PBS 5.5, 2 × 10^−3^ m Vit C condition, suggesting that reductant may play more critical role than hydrogen ion in intracellular ferric ions release. Altogether, we demonstrated that ETP‐PtFeNP nanoparticles were degradable and the release of oxaliplatin and ferric ions were both redox‐triggered and controlled.

α‐Enolase is a plasminogen‐binding receptor, which has been widely demonstrated to be overexpressed on the cell surface of most tumors (like colon cancer, pancreatic cancer, and breast cancer). The expression of α‐enolase usually correlates with the tumor diagnosis, survival, and prognosis. Moreover, the α‐enolase targeting peptide, ETP (SSMDIVLRAPLM), has been already reported as a promising tumor targeting moiety in many types of cancer therapies.^20^ We first explored the feasibility of employing α‐enolase as the targeting receptor by investigating the expression of α‐enolase in 4T1 tumor by q‐PCR analysis (Figure S26, Supporting Information). α‐Enolase was upregulated in 4T1 breast tumor tissues, compared with liver and kidney tissues. Nanoparticles were labeled with Cy5.5 for further optical investigation of cellular uptake efficiency in 4T1 cells. As shown in **Figure** **2**b, compared with PtFeNP group, an enhanced red fluorescence signal of Cy5.5 was observed in ETP‐PtFeNP group, suggesting that ETP modification could somehow improve the cellular uptake of nanoparticles by 4T1 cancer cells. Moreover, we found that the uptake behavior would be inhibited by pretreatment with 100‐fold free ETP, which indicated that the uptake enhancement of ETP‐PtFeNP was induced by the specific recognition between modified targeting moiety and α‐enolase. Similar results were also obtained by flow cytometer. To further investigate the intracellular distribution of ETP‐PtFeNP, a green‐fluorescent lysosomal probe was used. Upon a 15 min incubation, the red fluorescence of Cy5.5 showed colocalization with the green fluorescence of lysosome probe, while reduced colocalization was found after 1 h incubation (Figure 2c), showing that the nanoparticles might escape from the lysosome, which was probably due to the disruption of lysosomal membrane via the enhanced Fenton' reaction mediated by the released oxaliplatin and ferric ions.^16^

**Figure 2:**
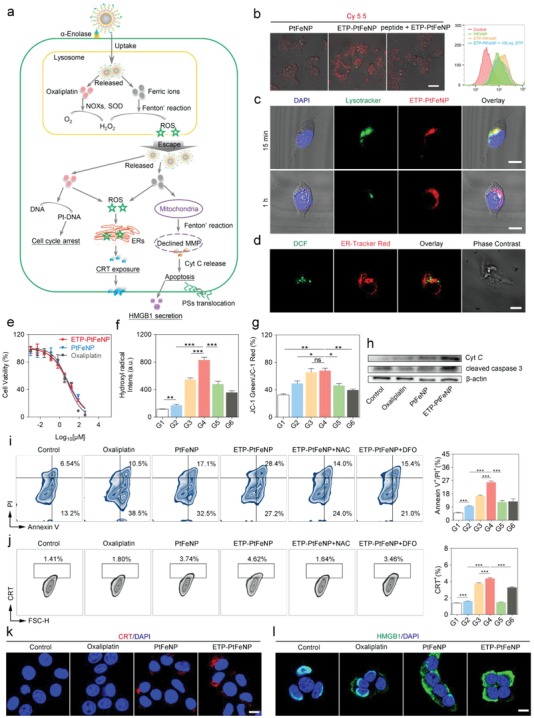
In vitro study of ETP‐PtFeNP formulations. a) Illustration of ETP‐PtFeNP modulation of three pathways for ICD induction. b) Cellular uptake of the Cy5.5‐labeled formulations on 4T1 cells by CLSM and flow cytometry analysis, respectively. Scale bar: 50 µm. c) Colocalization of ETP‐PtFeNP and lysosome in 4T1 cells under CLSM after 15 min treatment, and a declined colocalization was found after 1 h treatment. Scale bars: 10 µm. d) Colocalization of ROS (detected by DCF) and endoplasmic reticulum was found in 4T1 cells under CLSM. Scale bar: 10 µm. e) MTT assay of 4T1 cells after treated with various concentrations of different formulations for 48 h. Data are presented as means ± SD (*n* = 4). f) Hydroxyl radical generation. Data are presented as means ± SD (*n* = 4). g) The ratio of JC‐1 green to JC‐1 red fluorescence of 4T1 cells with different formulations treatment. Data are presented as means ± SD (*n* = 4). h) Western blotting analysis of Cyt C and cleaved caspase‐3 in 4T1 cells. i) Apoptosis assay of 4T1 cells by flow cytometry (left) and the relative quantification results (right) in different groups. Data are presented as means ± SD (*n* = 3). j) Flow cytometric analysis of CRT exposure (left) and the relative quantification results (right) in different groups. Data are presented as means ± SD (*n* = 3). k) CRT exposure and in 4T1 cells, following by CLSM. Scale bar: 10 µm. l) HMGB1 secretion in 4T1 cells, following by CLSM. Scale bar: 10 µm. G1: Control, G2: Oxaliplatin, G3: PtFeNP, G4: ETP‐PtFeNP, G5: ETP‐PtFeNP+NAC, G6: ETP‐PtFeNP+DFO. Significance is defined as ns, no significance, *P < 0.05, **P < 0.01, ***P < 0.001.

In vitro cytotoxicity of ETP‐PtFeNP, PtFeNP and oxaliplatin was investigated on 4T1 cells by MTT assay. As shown in Figure 2f, the inhibition of cell viability was concentration‐dependent, and the IC_50_ values of ETP‐PtFeNP, PtFeNP, and oxaliplatin were 7.209, 8.198, and 6.594 × 10^−6^ m, respectively, indicating that loading oxaliplatin into the nanoparticles as a prodrug form did not reduce its cytotoxicity. Meanwhile, since oxaliplatin is a cell cycle inhibitor which can suppress cell proliferation at G2/M phase, the results of cell cycle arrest experiment also confirmed that the prodrug‐loaded nanoparticles still processed the capability of cell cycle interruption (Figure S27, Supporting Information).

It has been reported that the internalized oxaliplatin can induce an intracellular H_2_O_2_ generation,^21^ via NOXs activation and SODs‐mediated superoxide anion (O_2_^·−^) dismutation. Fortunately, H_2_O_2_ also is the substrate of Fenton's reaction. To monitor the ROS level within cells, a fluorescent probe, 2′,7′‐dichlorofluorescein diacetate (H_2_DCFH‐DA) was adopted. As demonstrated in Figure 2g, oxaliplatin could induce a higher ROS level in 4T1 cells than control group, and the ROS level was further improved when treated with oxaliplatin (IV)‐loaded Fe_3_O_4_ nanoparticles, especially for the tumor‐targeting ETP‐PtFeNP. Furthermore, this amplification of ROS generation could be greatly inhibited by ROS scavenger, *N*‐acetyl‐*L*‐cysteine (NAC, 5 × 10^−3^ m) or iron chelator, deferoxamine mesylate (DFO, 100 × 10^−6^ m), which validated the involvement of ferric ions in this ROS enhancement. Additionally, we visualized that the generated ROS was partly localization with the ER (Figure 2e), which has been reported contribution to the ICD induction, via H_2_DCFH‐DA and ER‐Tracker Red staining. Considering these results above, we confirmed that tumor‐targeting modification and combination with the Fenton' effect of Fe_3_O_4_ nanoparticles could enhance the ROS generation of oxaliplatin, a classic type I ICD inducer.

In normal conditions, the mitochondria always maintain a high mitochondrial membrane potential (MMP) to produce ATP. However, when the intracellular oxidative stress is increased by external interference, a subsequent irreversible damage to mitochondria will be induced, resulting in MMP decrease and cytochrome C (Cyt C) release, as well as apoptotic cascade activation.^22^ Thus, MMP sensor JC‐1 was applied to evaluate the destruction of mitochondria after treatment. The average ratio of JC‐1 green to JC‐1 red fluorescence can be used as a probe of the MMP. As shown in Figure 2h, the ETP‐PtFeNP group showed the highest‐level mitochondrial membrane depolarization, while the ratio of green to red fluorescence shifted downward with the coincubation of NAC or DFO, exhibiting that the MMP decrease and mitochondrial damage were associated with ROS generation and ferric ions release. Similar results were also obtained by quantitative analysis with flow cytometry (Figure S28, Supporting Information).

Next, western blotting was performed to investigate the protein expression of Cyt C and cleaved caspase‐3 in the cells treated with oxaliplatin, PtFeNP, ETP‐PtFeNP, and Hank's solution as control. The expression of Cyt C and cleaved caspase‐3 increased in the order of control < oxaliplatin < PtFeNP < ETP‐PtFeNP (Figure 2i). Combining with the above findings in MMP measurement, this result indicated that ETP‐PtFeNP could induce apoptosis via ROS/Cyt C/caspase‐3 pathway. Besides we also evaluated the cell apoptosis by Annexin V‐FITC/propidium iodide (PI) staining assay. As shown in Figure 2k, the percentage of Annexin V/PI‐positive cells were increased after treatment with the ETP‐PtFeNP, which would also be inhibited by the addition of NAC or DFO. This was in consistency with the results of mitochondria disruption and indicated an ROS and ferric ion‐associated cell apoptosis by ETP‐PtFeNP.

As described by previous studies, CRT is one of the most abundant proteins in the ER and tends to be translocated to the surface of plasma membrane when ROS‐based ER stress occurs. On the other hand, HMGB1 is an abundant nuclear nonhistone chromatin‐binding protein which will be released from the nuclear when cell is dying. The CRT exposure and HMGB1 release are considered as surrogate markers for ICD‐associated immunogenicity, which can function as “eat me signals” for the immune system to recognize and process by antigen‐presenting cells, followed by T lymphocyte‐mediated adaptive immunity.[[qv: 2c]] We assessed the CRT exposure and HMGB1 release in 4T1 cells by immunofluorescence staining after short‐term stimulation (4 h) with oxaliplatin, PtFeNP and ETP‐PtFeNP, followed by CLSM or flow cytometric analysis. As illustrated in Figure 2j, the largest number of CRT staining cells were detected in the ETP‐PtFeNP group, suggesting that ETP‐PtFeNP treatment could stimulate the most translocation of CRT from the ERs to cell surface. Meanwhile, the CRT exposure could be hampered by addition of NAC or DFO, which indicated that the CRT exposure was ROS‐ and ferric ion‐dependent. Moreover, the strongest red fluorescence was detected by CLSM in the ETP‐PtFeNP group (Figure 2k), and ETP‐PtFeNP treatment could also induce an obviously increased HMGB1 release from the nuclei to the cytosol in 4T1 cells (Figure 2l). Additionally, the increased HMGB1 release from the ETP‐PtFeNP‐treated cancer cells into the extracellular fluid was also detected via western blot analysis of cell culture supernatant (Figure S29, Supporting Information).

Next, to investigate whether the enhanced DAMPs exposure from cancer cells treated with ETP‐PtFeNP can induce stronger immune responses than others, bone marrow‐derived DCs (treated with interleukin‐4 and granulocyte macrophage colony stimulating factor for 5 days after isolation) were incubated with conditioned supernatants from different formulations‐treated 4T1 cells for another 24 h. CD80 and CD86 were two biomarkers of DCs maturation.^24^ As shown in Figure S30 of the Supporting Information, significantly elevated expression of both CD80 and CD86 on DCs was induced by the cell culture medium from ETP‐PtFeNP treated 4T1 cells when compared to those of others, suggesting a reinforced DCs maturation in ETP‐PtFeNP treated group. Altogether, the combined results revealed that ETP‐PtFeNP could systematically synergize the efficacy of ferroptosis with chemotherapy to improve the intracellular ROS levels and result in mitochondrial damage and ER stress, eventually inducing enhanced immunogenic apoptosis, DAMPs exposure, and DC maturation.

Encouraged by the tumor targeting ability in vitro, we further examined the tumor targeting efficiency and tissue distribution of ETP‐PtFeNP or PtFeNP (as control) in the 4T1 breast tumor‐bearing mice, via tail‐vein injection of Cy5.5‐labeled nanoparticles and detection by Xenogen IVIS Spectrum CT instrument. Notably, the mice receiving tumor‐targeting nanoparticles, ETP‐PtFeNP, exhibited stronger near‐infrared (NIR) signal at the tumor sites (indicated with the arrow) than the ones treated with PtFeNP, at 12 h or 24 h after intravenous injection (**Figure** **3**a). Mice were sacrificed 24 h after injection. The ex vivo biodistribution of Cy5.5‐labled nanoparticles was further assessed and quantified by IVIS. As shown in Figure 3b,c, a higher NIR signal at the tumor site was detected in the targeting group, showing enhanced active tumor targeting ability with the modification of ETP, through α‐enolase mediated tumor binding and internalization. Interestingly, a lower NIR signal was found in the liver of the targeting group. This phenomenon may also be attributed to the tumor‐targeting capacity of ETP modification that enhanced accumulation of nanoparticles at tumor sites, accordingly decreasing nanoparticles‐distribution in blood circulation and leading to a lower NIR signal at the liver. To further evaluate the intratumoral distribution of nanoparticles, we semiquantified the Cy5.5‐labeled nanoparticles in the sections of tumors by confocal imaging. A rabbit polyclonal antibody against CD34 and an Alexa Fluor 488‐conjugated secondary antibody were used to stain the blood vessel in the tissue sections. In the tumor tissues where a similar distribution of blood vessels was presented, the detected Cy5.5 fluorescence of ETP‐PtFeNP treated group was stronger than that of PtFeNP treated group (Figure 3d; Figure S31, Supporting Information). Summing up the above results, the ETP‐PtFeNP formulation showed an excellent tumor‐targeting effect, as one of the essential requirements for efficient antitumor therapy.

**Figure 3:**
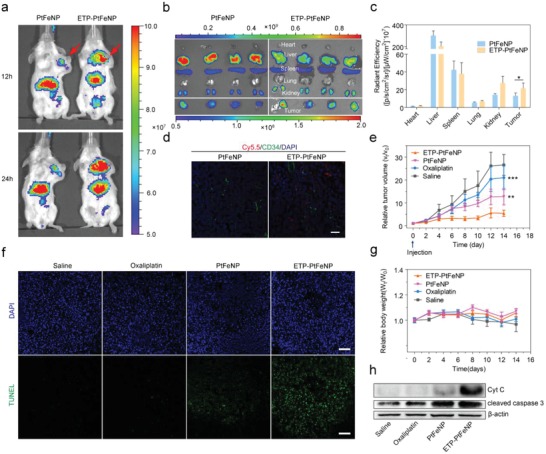
In vivo study of ETP‐PtFeNP formulations. a) In vivo images of tumor‐bearing mice intravenously administrated with Cy5.5‐labeled formulations at 12 and 24 h post the injection by IVIS. b) Ex vivo images of excised organs isolated from tumor‐bearing mice by IVIS at 24 h post the injection. c) Biodistribution of the Cy5.5‐labeled nanoparticles 24 h after intravenous injection into tumor‐bearing mice (*n* = 4). d) CD34‐staining and nanoparticle distribution in frozen tumor sections from tumor‐bearing mice at 24 h after administration with Cy5.5‐labeled nanoparticles. Scale bar: 30 µm. e) Tumor volume change and g) body weight change of 4T1 breast tumor‐bearing mice after intravenously injected with different oxaliplatin formulations. Data are presented as means ± SD (*n* = 6). f) TUNEL assay of 4T1 tumor xenografts excised from mice models. Scale bars: 100 µm. h) Western blotting analysis of Cyt C and cleaved caspase‐3 expression in 4T1 tumor xenografts tissues. Significance is defined as *P < 0.05, **P < 0.01, ***P < 0.001.

We then assessed the therapeutic efficacy of ETP‐PtFeNP compared with PtFeNP or oxaliplatin in the 4T1 tumor‐bearing balb/c mice, following the therapeutic schedule that intravenous administration was performed every three days for five times (oxaliplatin: 5 mg kg^−1^), while saline was used for the control group. For instance, Huang reported a low drug dose of 6 mg kg^−1^ in immune‐associated therapy.^23^ We adopted a drug dose as low as 5 mg kg^−1^, due to the low drug dose, no obvious tumor growth suppression was observed in the oxaliplatin treated group (Figure 3e; Figure S32, Supporting Information). By contrast, the ETP‐PtFeNP treatment exhibited a significant antitumor effect compared with other groups. Besides, there was no evident weight loss during the experiments (Figure 3g). We subsequently carried out the TUNEL staining and western blot analysis of Cyt C and cleaved caspase‐3 expression in the tumor tissue after treatments. Both results clearly showed that ETP‐PtFeNP could lead to a Cyt C/cleaved caspase‐3 pathway‐based irreversible cell apoptosis (Figure 3f,h), which was expected to contribute to further DAMPs exposure and elicitation of tumor‐specific immune responses.

Next, we investigated the role of immune system in antitumor treatment of ETP‐PtFeNP by analyzing the cell components in the single cell suspensions of tumor draining lymph nodes (TDLN) or tumor tissues isolated from the treated mice by flow cytometry. CD80 and CD86 were two biomarkers of DCs maturation.^24^ Compared with the control group, mice treated with oxaliplatin, PtFeNP, or ETP‐PtFeNP, were all observed with an increased number of mature CD80^+^ CD86^+^ DCs in TDLN (**Figure** **4**a), especially, the ETP‐PtFeNP group showed the highest increase. In addition, the increased CRT exposure and enhanced HMGB1 release were also both confirmed by CRT staining of tumor tissue sections and western blot analysis of CRT and HMGB1 expressions (Figure 4e,g). These results might be mainly owing to the effective accumulation of ETP‐PtFeNP in tumor regions, which stimulated cancer cells to undergo an immunogenic apoptosis and promoted the exposure of DAMPs, the critical stimulus for DC maturation. The activated DCs could further promote the recruitment and differentiation of T lymphocytes around the tumor tissues. As shown in Figure 4b, compared with control group, the amount of cytotoxic CD8^+^ and helper CD4^+^ T cells were significantly increased in the ETP‐PtFeNP group, suggesting the enhancement of adoptive antitumor immune responses.^25^ Furthermore, we found that most of increased CD4^+^ T cells were CD4^+^ CD25^−^ T cells (Figure 4c), termed as effector T cells, which act as immune promoters in immune inflammatory responses.^26^ On the other hand, the percentage of immunosuppressive CD4^+^ CD25^+^ regulatory T cells among CD4^+^ T cells were declined after oxaliplatin, PtFeNP and ETP‐PtFeNP treatments. In addition, increased percentages of CD8^+^ and CD4^+^ T cells were also both found in splenocytes isolated from mice treated with ETP‐PtFeNP (Figure S35, Supporting Information), revealing that entire body immunity might have been activated to defense against cancer. Meanwhile, an up‐regulated secretion of IFN‐γ in tumor tissues was detected in ETP‐PtFeNP group (Figure 4d), as a critical factor for innate and adaptive immunity against tumors.^[25]^ Moreover, it has been reported that the tumor immunosuppression was also induced by the interaction between overexpressed PD‐L2 and PD‐1 in tumor tissues.[[qv: 8a]] As demonstrated, PD‐L2 was highly expressed in the established 4T1 breast tumor, and a downregulation of PD‐L2 expression was observed after the treatment with oxaliplatin‐contained formulations (Figure 4f,h), indicating PD‐L2 and PD‐1 interaction‐mediated immunosuppression might be alleviated after treatment with oxaliplatin‐contained formulations. From the above, the tumor‐targeting formulation, ETP‐PtFeNP, could not only induce a significant immunogenic cell death, but also reverse the PD‐L2 mediated immunosuppression in tumor microenvironment, which synergistically elicited an effective antitumor immune response to enhance the inhibition of tumor growth *in vivo*.

**Figure 4:**
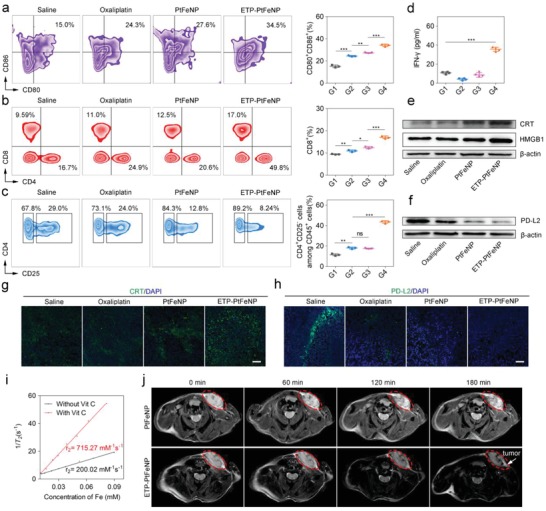
Enhanced immunity and reinforced MRI effect. a) Representative plots of DCs (left, gated on CD11c^+^ cells) and the relative quantification results (right) in tumor draining lymph nodes of the 4T1 breast tumor‐bearing mice after various treatments. Data are presented as means ± SD (*n* = 3). b,c) Representative plots of T cells (left, gated on CD45^+^ cells) and the relative quantification results (right) in treated tumor analyzed by flow cytometry. Data are presented as means ± SD (*n* = 3). d) IFN‐γ levels in 4T1 breast tumors at day 18 after mice received the first indicated treatment. Data are presented as means ± SD (*n* = 4). e) Western blotting analysis of CRT and HMGB1 expression in 4T1 breast tumor xenografts tissues. f) Western blotting analysis of PD‐L2 expression in 4T1 breast tumor xenografts tissues. g,h) Representative image of immunofluorescence staining of the tumor sections showing increased CRT exposure and declined PD‐L2 expression. Scale bars: 10 µm. i) *T*_2_ relaxation rate (1/*T*_2_) as a function of Fe concentration in ETP‐PtFeNP nanoparticles that were incubated with (red) or without (black) Vit C. j) *T*_2_‐weighted MR images recorded for mice bearing 4T1 breast tumor. G1: Control, G2: Oxaliplatin, G3: PtFeNP, G4: ETP‐PtFeNP. Significance is defined as ns, no significance, *P < 0.05, **P < 0.01, ***P < 0.001.

In general, a favorable *T*_2_ contrast agent should have the following two characteristics: a suitable particle size, within limits, smaller particles are preferred for in vivo application as they generally have a longer blood half‐life than larger counterparts; a higher *T*_2_‐weighted transverse relaxivity (*R*_2_), which is beneficial for reducing the dose of contrast agent. However, the traditional method to enhance the *T*_2_ effect of contrast agents is usually by the increase of particle size, which in turn impacts their biological half‐life.^27^ Based on this, stimuli‐induced aggregating magnetic nanoparticles may meet these requirements well. Therefore, we first recorded the *T*_2_‐weighted spin–spin MR images of ETP‐PtFeNP nanoparticles in aqueous solution. As shown in Figure 4i, the *T*_2_ relaxation rate (1/*T*_2_) increased linearly with the Fe concentrations (black line) and the calculated *R*_2_ value of ETP‐PtFeNP was 200.02 mm ^−1^ s^−1^, which suggested that ETP‐PtFeNP could be used as a fine *T*_2_‐shortening agent due to its small size and large *R*_2_ value. Encouragingly, the *R*_2_ value of nanoparticles was increased by more than three times, up to 715.27 mm ^−1^ s^−1^, due to the aggregation of nanoparticles over 3 h incubation with 2 × 10^−3^ m Vit C. The redox‐triggered aggregation might enhance the MRI negative contrast effect in the reductive intracellular tumor environment where ETP‐PtFeNP was accumulated.

The favorable in vitro performance of ETP‐PtFeNP as an MRI contrast agent drove us to pursue their applicability for in vivo applications. *T*_2_‐weighted MRI of the tumor site was darkened after 180 min intravenous administration of ETP‐PtFeNP (Figure 4j). Meanwhile, the MRI negative contrast effect of PtFeNP was inconspicuous without effective tumor accumulation. Due to the active tumor‐targeting and redox‐triggered aggregation capabilities, ETP‐PtFeNP may have the potential to act as a high‐sensitivity *T*_2_ negative contrast agent for MRI in the future.

H&E staining results showed no pathological abnormalities in the major organs between control and treated groups (Figure S38, Supporting Information), showing the biosafety of the formulations. Furthermore, ototoxicity is a serious side effect of Pt‐based drugs, which should be paid more attention to when applied in clinic.^28^ Hearing test results also indicated that loading oxaliplatin into a nanocarrier could alleviate the hearing impairment (Figure S39, Supporting Information).

In summary, a novel nanoplatform ETP‐PtFeNP was constructed for reinforced tumor‐targeting ICD. The nanoplatform can elicit effective antitumor immune responses by actively accumulating in tumors, enhancing ICD induction, reinforcing DCs maturation, reversing immunosuppression, and activating antitumor T cells. In particular, ETP‐PtFeNP can increase the accumulation of drugs at tumor sites through EPR effect and ETP‐mediated active tumor‐targeting. Released ferric ions can synergistically boost the ROS generation effect of released oxaliplatin, resulting in reinforced ICD. Afterward, exposed DAMPs can facilitate the DCs maturation and lead to cross‐presentation of tumor antigens and antitumor specific T‐cell responses. Meanwhile, released oxaliplatin can further reverse PD‐L2 mediated immunosuppression. Therefore, a comprehensive inhibition of tumor growth was achieved. Moreover, the *T*_2_‐weight MRI effect of ETP‐PtFeNP was remarkably improved while triggered by reductant. Finally, these above advantages make ETP‐PtFeNP a promising, multimodal agent for image‐guided immunogenic chemotherapy.

## Conflict of Interest

The authors declare no conflict of interest.

## Supporting information

Supplementary
