# Supplementary material for: Tumor Microenvironment‐Triggered Aggregated Magnetic Nanoparticles for Reinforced Image‐Guided Immunogenic Chemotherapy
Source: Adv Sci (Weinh). 2019 Jan 29;6(6):1802134. doi: 10.1002/advs.201802134 (PMC6425432; doi:10.1002/advs.201802134)
Supplement: Supplementary file 1 — Supplementary [file ADVS-6-1802134-s001.pdf]

Copyright WILEY-VCH Verlag GmbH & Co. KGaA, 69469 Weinheim, Germany, 2019.

ADVANCED SCIENCE
Open Access

Supporting Information

for *Adv. Sci.,* DOI: 10.1002/advs.201802134

Tumor Microenvironment-Triggered Aggregated Magnetic Nanoparticles for Reinforced Image-Guided Immunogenic Chemotherapy

*Qinjun Chen, Lisha Liu, Yifei Lu, Xinli Chen, Yujie Zhang, Wenxi Zhou, Qin Guo, Chao Li, Yiwen Zhang, Yu Zhang, Donghui Liang, Tao Sun, and Chen Jiang**

Copyright WILEY-VCH Verlag GmbH & Co. KGaA, 69469 Weinheim, Germany, 2018.

Supporting Information

Tumor Microenvironment-Triggered Aggregated Magnetic Nanoparticles for Reinforced Image-Guided Immunogenic Chemotherapy

Qinjun Chen, Lisha Liu, Yifei Lu, Xinli Chen, Yujie Zhang, Wenxi Zhou, Qin Guo, Chao Li, Yiwen Zhang, Yu Zhang, Donghui Liang, Tao Sun, and Chen Jiang*

Contents:

Scheme S1. Synthesis route of ETP-OXA-DHAC or PEG-OXA-DHAC polymer

Figure S1-S17. ^{1}H NMR, MS-ESI, IR and GPC characterization of compounds

Figure S18. HPLC characterization of ETP-OXA-DHAC polymer

Figure S19. TEM images of oleic acid-Fe_3O_4

Figure S20. Digital photographs depict the hydrophilicity of Fe_3O_4 nanoparticles after modification

Figure S21. Standard curve of oxaliplatin by HPLC

Figure S22-S23. HPLC spectra of triggered oxaliplatin release from ETP-PtFeNP nanoparticles

Figure S24. Photographs and corresponding DLS data of serveral core-shell Fe_3O_4 aqueous dialysis solutions

Figure S25. Standard curve of Fe^{2+} by Automatic microplate reader at UV 512 nm

Figure S26. Q-PCR measuring the α-enolase mRNA expression

Figure S27. Cell cycle analysis

Figure S28. Flow cytometric analysis of mitochondrial membrane damage

Figure S29. Western blotting analysis of HMGB1 release

Figure S30. Flow cytometric analysis of DCs maturation

Figure S31. Semi-quantitative analysis of the Cy5.5-fluorescence in tumor sections

Figure S32. Representative tumor images

Figure S33-S34. Gating strategy for immune cells' frequencies determination

Figure S35. Representative plots of T cells in spleen

Figure S36-S37. T_2 relaxation rate ($1/T_2$) as a function of Fe concentration in several magnetic nanoparticles solution

Figure S38. H&E staining of main organs

Figure S39.Schematic diagram of instruments for detecting audiometric thresholds

Table S1. Antibody information

Experimental section

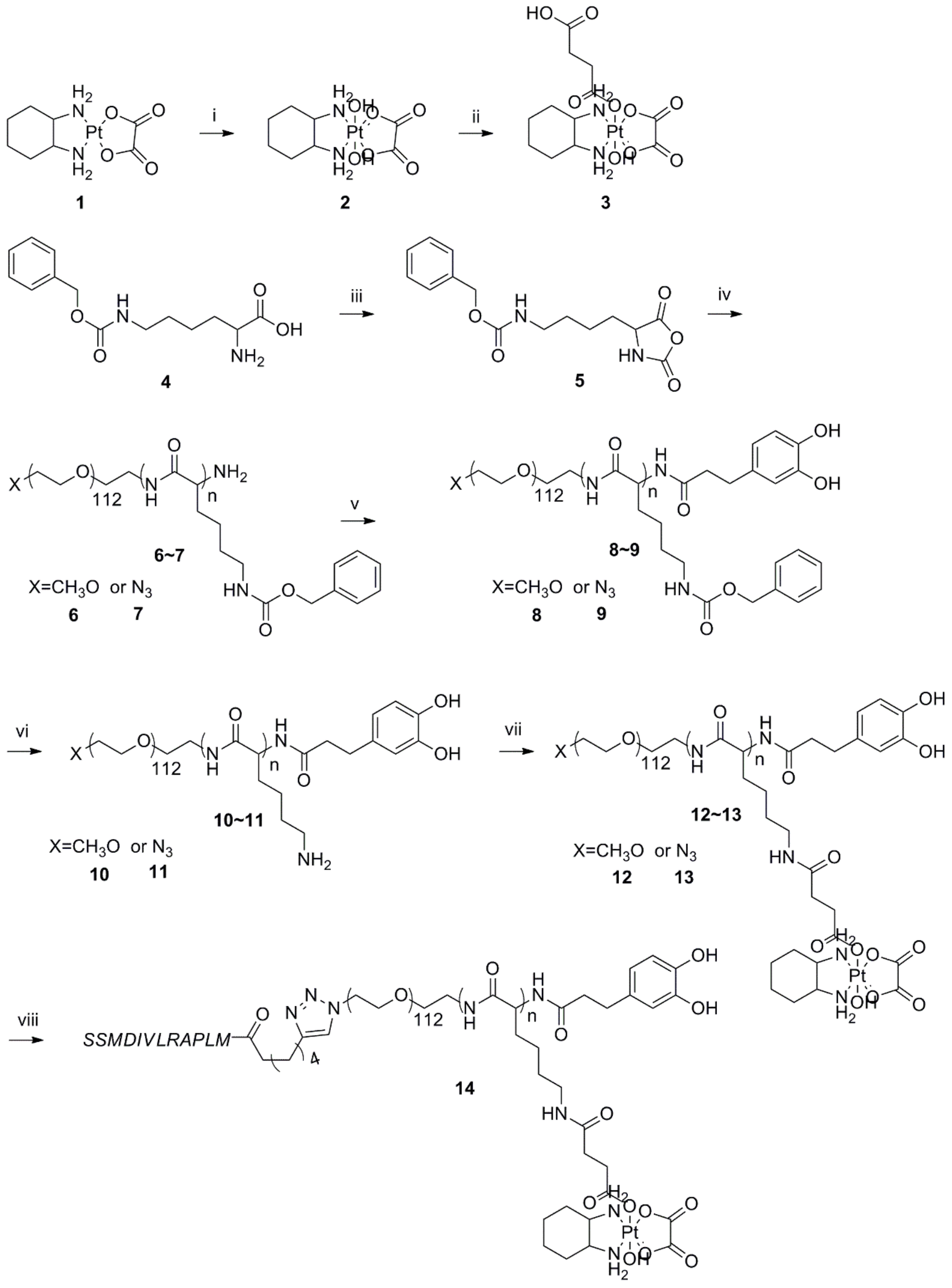

Scheme S1. Synthetic steps and structures of ETP-OXA-DHAC or PEG-OXA-DHAC polymer.

Detailed steps as shown below:

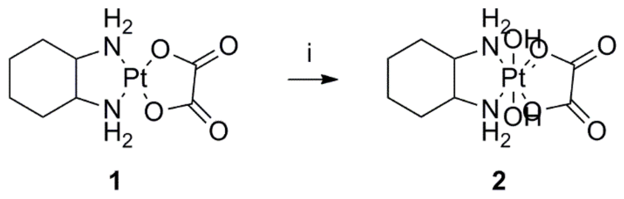

This reaction was followed by the reported steps. Oxaliplatin (**1**, 200 mg, 0.5 mmol) was suspended in H_2O_2 (30% aqueous solution, 10 mL) at dark and stirred at r. t. for 12 h. The whole mixture was freeze-dried to give 2 as a yellowish powder (216 mg, 100%).

Rf = 0.7 (DCM:methanol=10:1, v:v).

^{1}H NMR (400 MHz, D_2O, δ, ppm): 2.70-2.57 (m, 2H, H-1, 10), 2.12-2.02 (m, 2H, H-2, 8), 1.50-1.29 (m, 4H, H-4~7), 1.12-1.00 (m, 2H, H-3, 9).

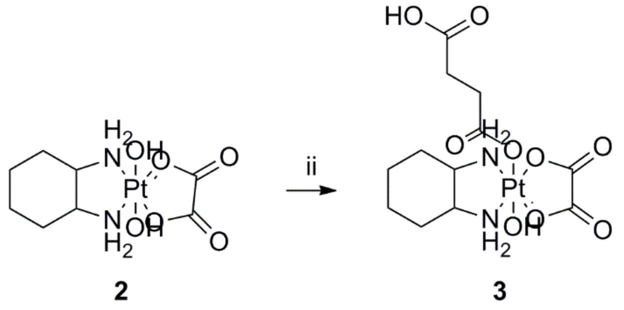

This reaction was followed by the reported steps. **2** (200 mg, 0.46 mmol) and anhydrous triethylamine (1 mg, 0.01 mmol) was suspended in anhydrous dimethyl sulfoxide (DMSO, 20 mL) and added with succinic anhydride (54 mg, 0.55 mmol). The mixture was maintained at dark/r. t. for 24 h. After cooling to r. t., the mixture was precipitated in Et_2O thrice to give **3** (244 mg, 100%) as a white powder.

Rf = 0.6 (DCM:methanol:acetic acid=10:1:0.01, v:v:v).

^{1}H NMR (400 MHz, D_2O, δ, ppm): 2.84-2.71 (m, 2H, H-1, 10), 2.60-2.45 (m, 4H, H-11~12), 2.28-2.15 (m, 2H, H-2, 8), 1.63-1.41 (m, 4H, H-4~7), 1.29-1.08 (m, 2H, H-3, 9).

MS-ESI Calc. for $C_{12}H_{21}N_2O_9Pt$ $[\mathbf{3}+H]^+$ 532.1 (100%), 531.1 (86.1%), 533.1 (80.7%), Found, 532.0, 531.0, 533.0; MS-ESI Calc. for $C_{12}H_{20}N_2HNaO_9Pt$ $[\mathbf{3}+Na]^+$ 554.1 (100.0%), 553.1 (85.8%), 555.1 (79.4%), Found, 554.0, 553.0, 555.0.

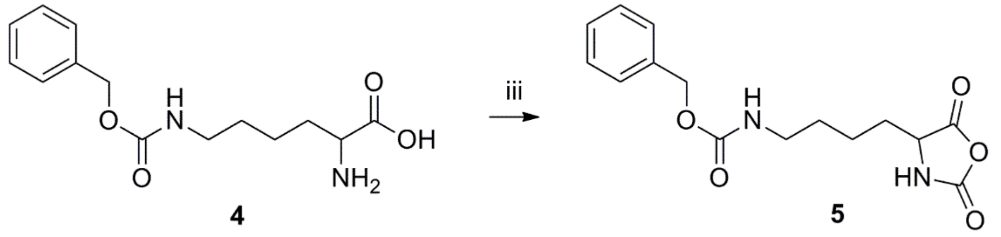

The protocol was followed by the previous literature. N^6-Carbobenzyloxy-L-lysine (**4**, 500 mg, 1.78 mmol) and triphosgene (211.6 mg, 0.71 mmol) was suspended in anhydrous tetrahydrofuran (30 mL). The mixture was maintained at 50 °C for 6 h under Ar. After cooling to r. t., the mixture was precipitated in n-Hexane thrice to give **5** (520 mg, 95%) as a white powder.

^{1}H NMR (400 MHz, DMSO-δ6, δ, ppm): 7.39-7.27 (m, 5H, H-1~5), 5.01-4.98 (m, 2H, H-6, 7), 4.44-4.39 (s, 1H, H-16), 3.02-2.93 (m, 2H, H-8,9), 1.80-1.20 (m, 6H, H-10~15).

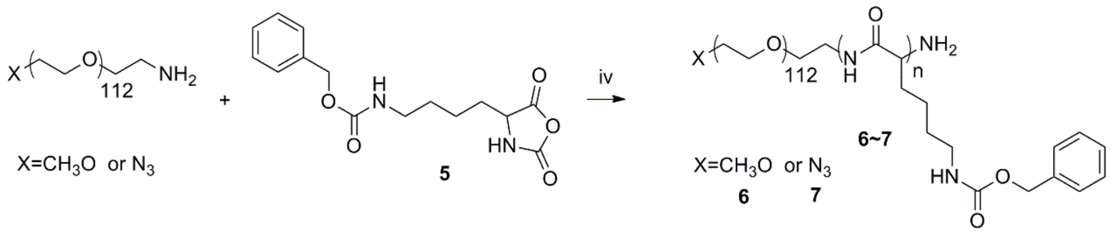

CH_3O-PEG_{5K}-NH_2 (Mw. 5,000) (500 mg, 0.1 mmol) and **5** (398 mg, 1.3 mmol) were dissolved in anhydrous DMSO (20 mL) and stirred at 50 °C for 72 h under Ar. After cooling to r. t., the mixture

was dialysis against H_2O for 48 h and freeze-dried to give **6** as a white powder. **7** was obtained through similar procedures except with N_3-PEG_{5K}-NH_2 (Mw. 5,000) as the stating material.

For **6**: ^{1}H NMR (400 MHz, DMSO-δ6, δ, ppm): 7.39-7.27 (m, 60H, H-1~5), 5.01-4.98 (m, 24H, H-6,7), 3.54-3.45 (m, 448H, H-protons of PEG), 3.25-3.23 (m, 3H, H-methoxy of PEG), 3.01-2.89 (m, 24H, H-8,9), 1.70-1.10 (m, 72H, H-10~15).

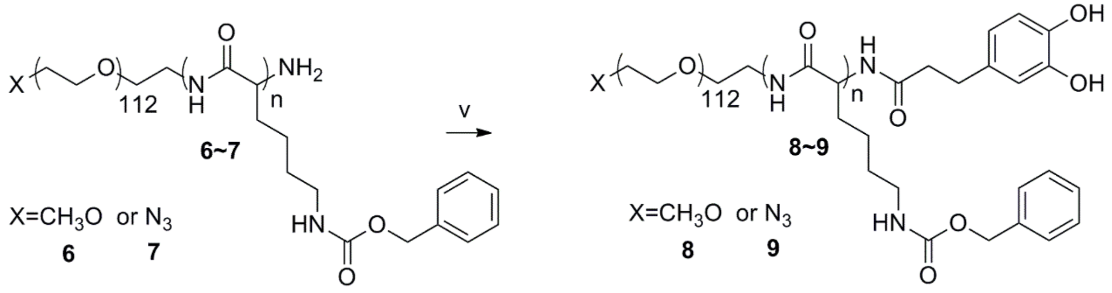

6 (500 mg, 0.06 mmol), 3,4-Dihydroxyhydrocinnamic acid (DHAC,21.8 mg, 0.12 mmol), 2-(7-Azabenzotriazol-1-yl)-*N,N,N',N'*-tetramethyluronium hexafluorophosphate (HATU, 45.6 mg, 0.12 mmol), 1-Hydroxybenzotriazole (HOBT, 16.2 mg, 0.12 mmol) and *N,N*-Diisopropylethylamine (DIPEA, 15.5 mg, 0.12 mmol) were suspended in anhydrous DMSO (20 mL) under Ar/r. t. for 24 h. the mixture was dialysis against H_2O for 48 h and freeze-dried to give **8** (CH_3O-PEG_{5K}-pLys (cbz)-DHAC) as a white powder. **9** (N_3-PEG_{5K}-pLys (cbz)-DHAC) was obtained through similar procedures.

For **8**: ^{1}H NMR (400 MHz, DMSO-δ6, δ, ppm): 7.39-7.27 (m, 60H, H-1~5), 6.63-6.39 (m, 3H, H-20~22), 5.01-4.98 (m, 24H, H-6,7), 3.54-3.45 (m, 448H, H-protons of PEG), 3.25-3.23 (m, 3H, H-methoxy of PEG), 3.01-2.89 (m, 24H, H-8,9), 1.70-1.10 (m, 76H, H-10~19).

For **9**: the GPC elution time was shifted from 7.46 min (N_3-PEG_{5K}-NH_2) to 6.93 min.

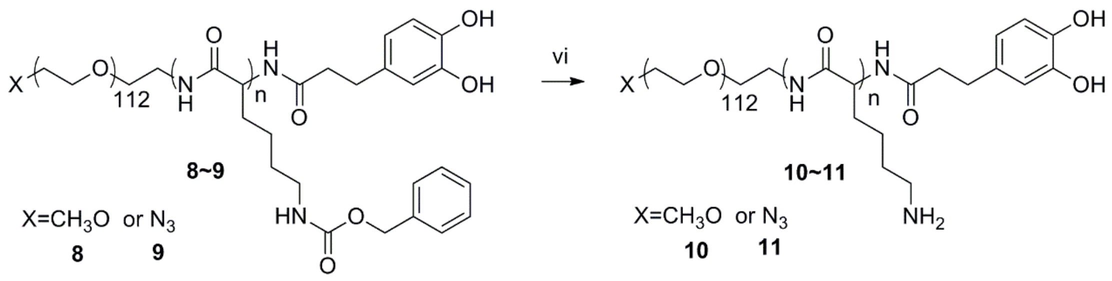

8 (500 mg, 0.06 mmol) was dissolved in trichloroacetic acid solution (TFA, 10 mL) at dark. HBr/HOAc (0.5 mL) was slowly added into the above solution and stirred at r. t. for 5 h. The mixture was precipitated in anhydrous ether (Et_2O) thrice and the crude was dialysis against H_2O for 48 h and freeze-dried to give **10** (CH_3O-PEG_{5K}-pLys (NH_2)-DHAC) as a white solid. **11** (N_3-PEG_{5K}-pLys (NH_2)-DHAC) was obtained through similar procedures.

For **10**: ^{1}H NMR (400 MHz, DMSO-δ6, δ, ppm): 6.63-6.39 (m, 3H, H-1~3), 3.54-3.45 (m, 448H, H-protons of PEG), 3.25-3.23 (m, 3H, H-methoxy of PEG), 2.83-2.69 (m, 18H, H-14,15), 1.70-1.10 (m, 60H, H-4~13).

For **11**: the GPC elution time was shifted from 6.93 min (**9**) to 7.42 min.

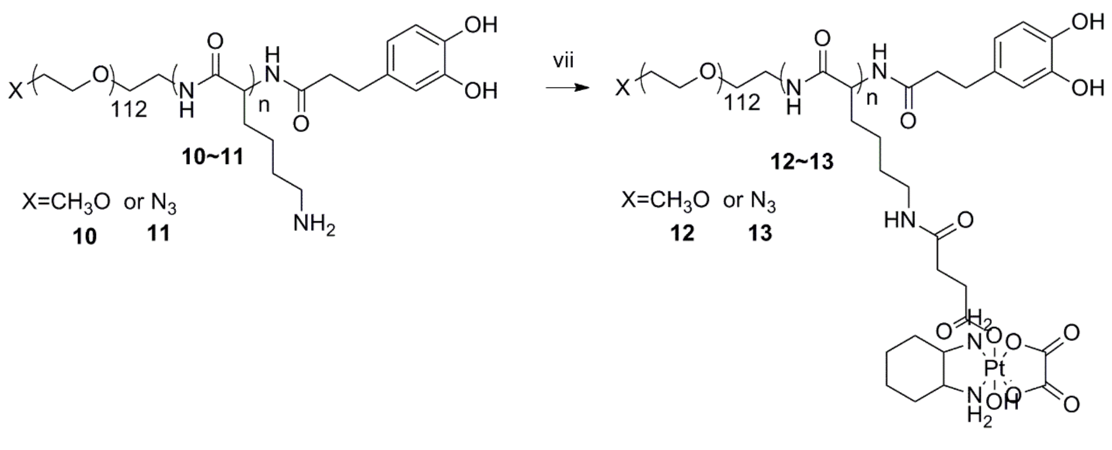

10 (200 mg, 0.03 mmol), **3** (111 mg, 0.21 mmol), HATU (79.8 mg, 0.21 mmol), HOBT (28.4 mg, 0.21 mmol) and DIPEA (15.5 mg, 0.12 mmol) were suspended in anhydrous DMSO (20 mL) under Ar/r. t. for 24 h. the mixture was dialysis against H_2O for 48 h and freeze-dried to give **12** (CH_3O-PEG_{5K}-OXA-DHAC) as a white powder. **13** (N_3-PEG_{5K}-OXA-DHAC) was obtained through similar procedures.

For **12**: ^{1}H NMR (400 MHz, DMSO-δ6, δ, ppm): 6.63-6.39 (m, 3H, H-1~3), 3.54-3.45 (m, 448H, H-protons of PEG), 3.25-3.23 (m, 3H, H-methoxy of PEG), 3.01-2.89 (m, 20H, H-14,15), 2.74-2.64 (m, 18H, H-20,29), 2.50-2.22 (m, 36H, H-16~19), 2.18-2.05(m, 18H, H-21,28),1.70-1.00 (m, 114H, H-4~3, 22~27).

For **13**: the GPC elution time was shifted from 7.42 min (**11**) to 5.30 min.

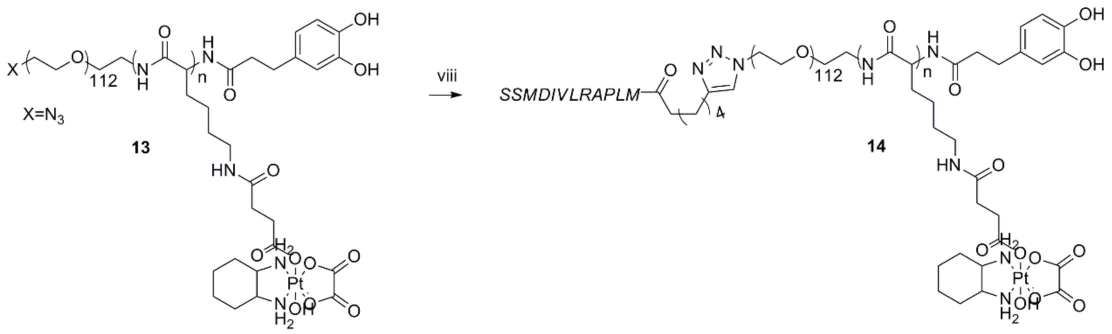

13 (50 mg, 0.005 mmol), *SSMDIVLRAPLM*-(5-Hexynoic acid) (14.3 mg, 0.01 mmol), CuI (2 mg, 0.01 mmol), and *N,N,N',N'',N''*-pentamethyldiethylenetriamine (PMDETA, 5 mg, 0.029 mmol) were suspended in anhydrous DMF (2 mL) under Ar/r. t. at dark overnight. The mixture was precipitated in Et2O thrice and the crude solid was dialysis against EDTA solution (100 mmol, 2 L) through a membrane (5K) and water respectively for 48 h, then freeze-dried to give **14** as a yellowish solid.

^{1}H NMR (400 MHz, DMSO-δ6, δ, ppm): 9.55-9.45 (s, H-triazole), 8.50-6.81 (m, H-amido bonds and aromatic rings), 6.63-6.39 (m, H-protons of dihydroxybenzene), 3.01-2.89 (m, H-protons of L-lysine (14,15)), 2.50-2.05 (m, H-protons of prodrug (20,29,16~19,21,28)), 1.70-1.00 (m, H-protons of L-lysine (4~13) and prodrug (22~27)), 0.90-0.75 (m, H-protons of peptide).

For **14**: the GPC elution time was shifted from 5.30 min (**13**) to 4.70 min.

FT-IR: as shown in **Figure S15**, the disappearance of azide group at 2100 cm^{-1} occurred after successful ETP peptide conjugation.

HPLC: as shown in **Figure S18**, the increasing peak area at 6.35 min in **d** indicated the successful synthesis of compounds (**14**).

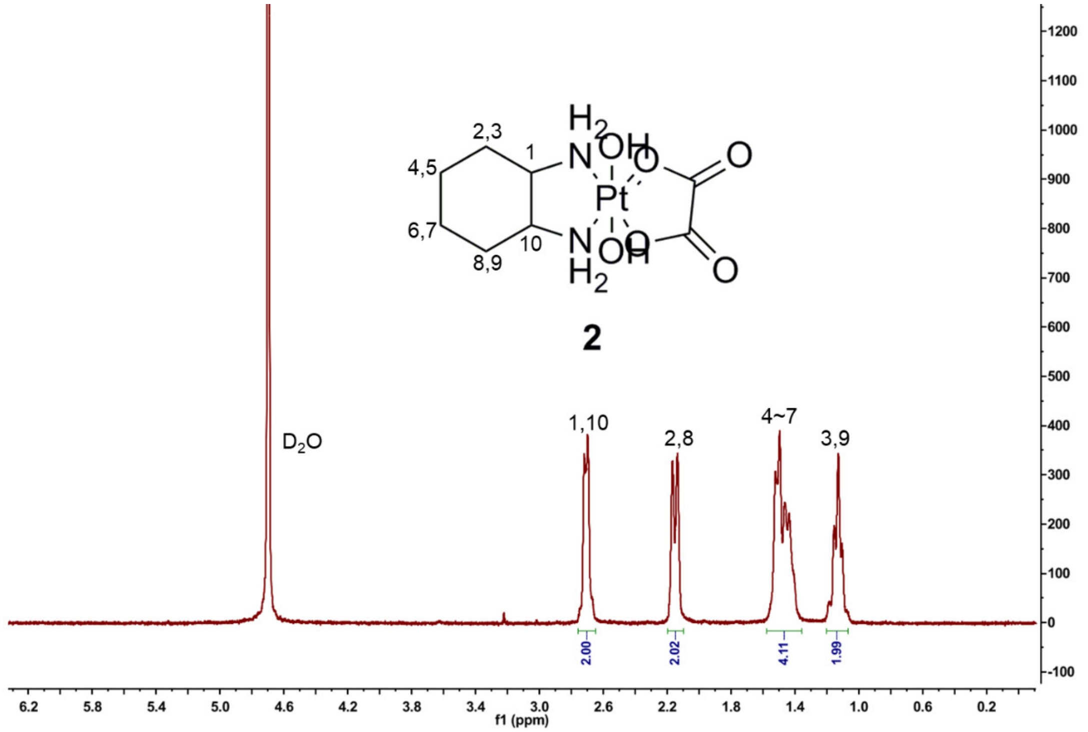

Figure S1. The ^{1}H NMR spectrum of **2** in D_2O.

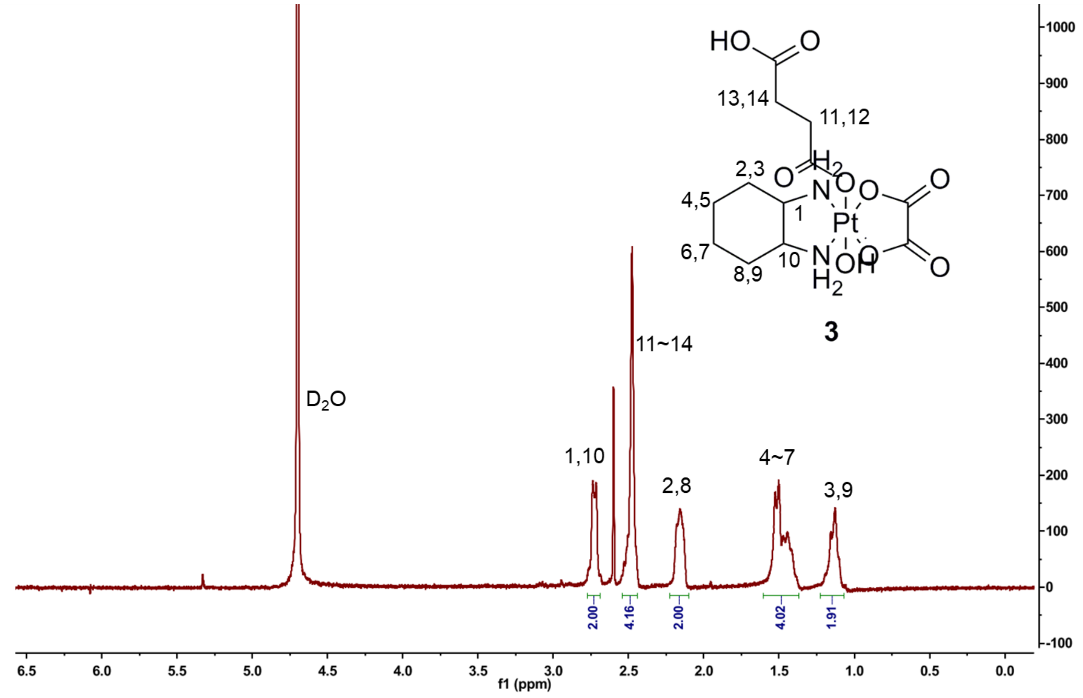

Figure S2. The ^{1}H NMR spectrum of **3** in D_2O.

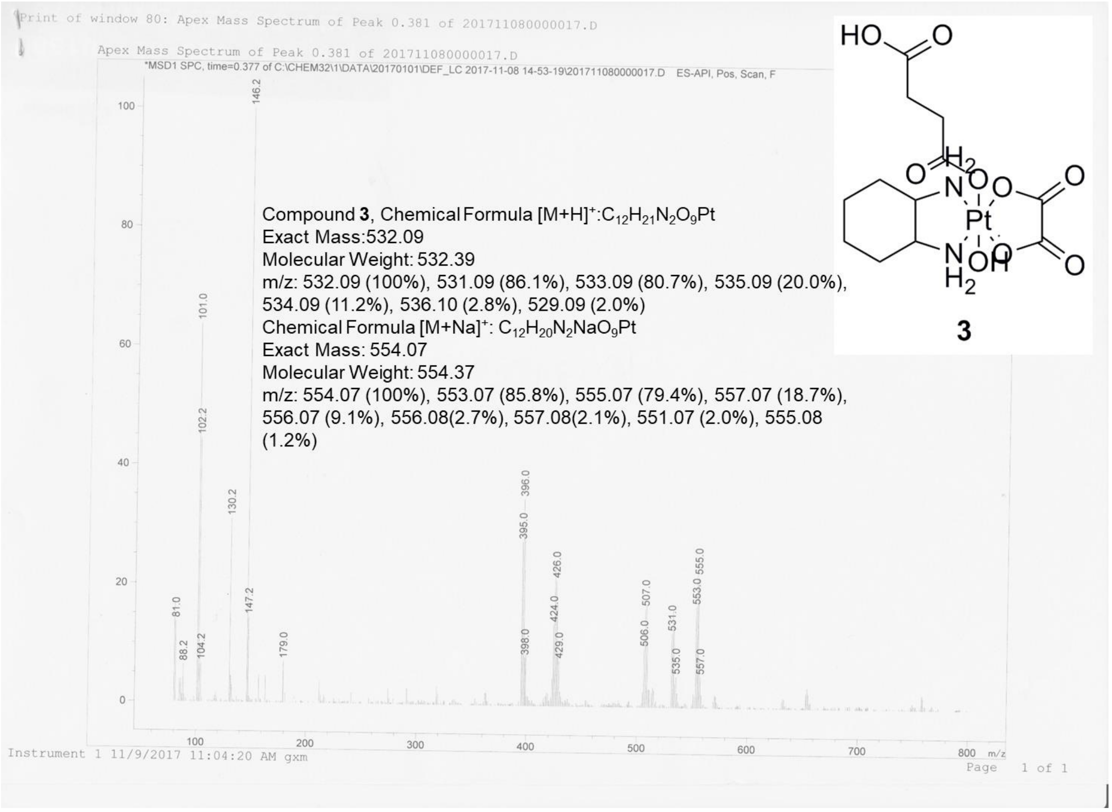

Figure S3. The MS-ESI spectrum of **3**.

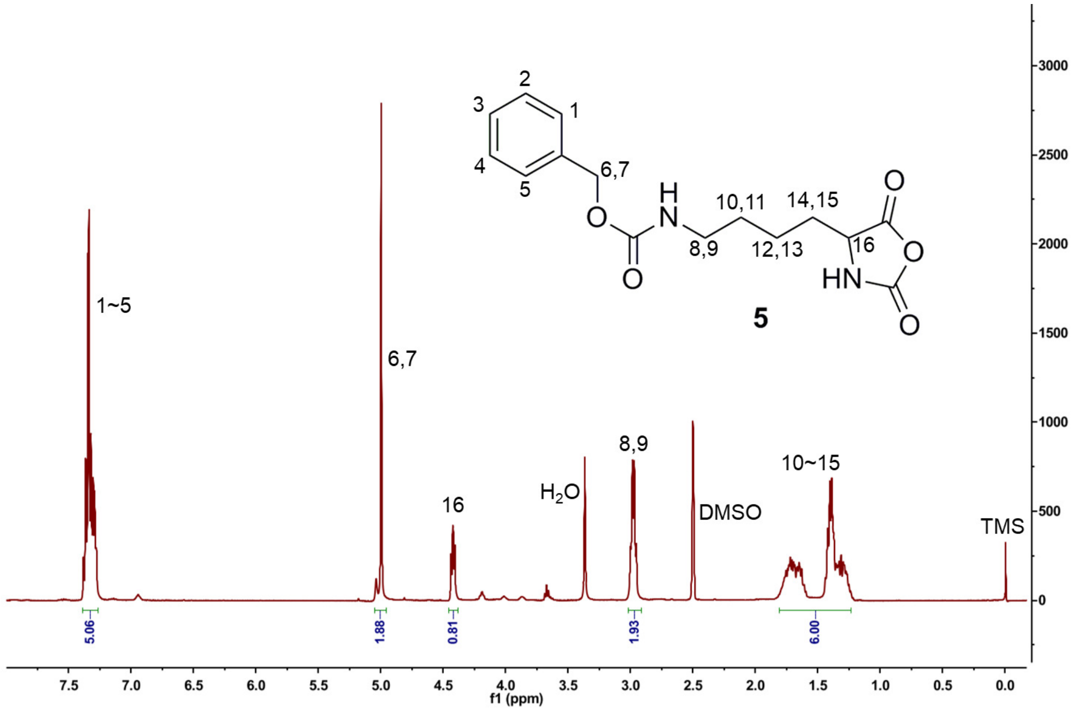

Figure S4. The ^{1}H NMR spectrum of **5** in DMSO-δ6.

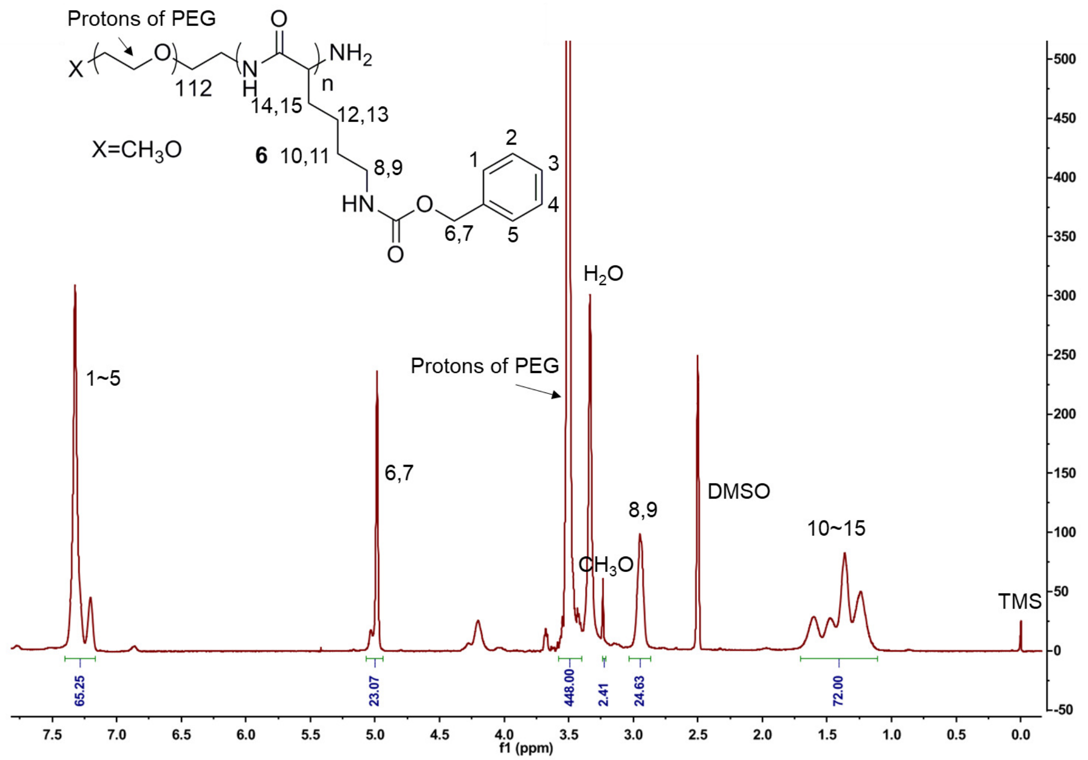

Figure S5. The ^{1}H NMR spectrum of **6** in DMSO-δ6.

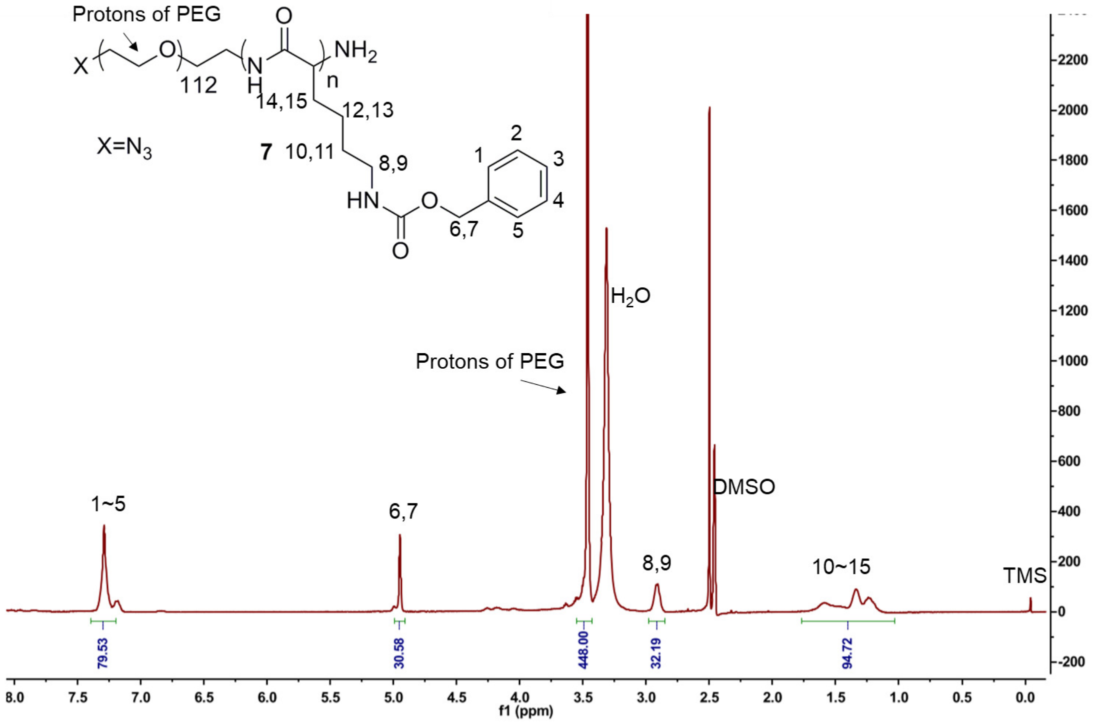

Figure S6. The ^{1}H NMR spectrum of **7** in DMSO-δ6.

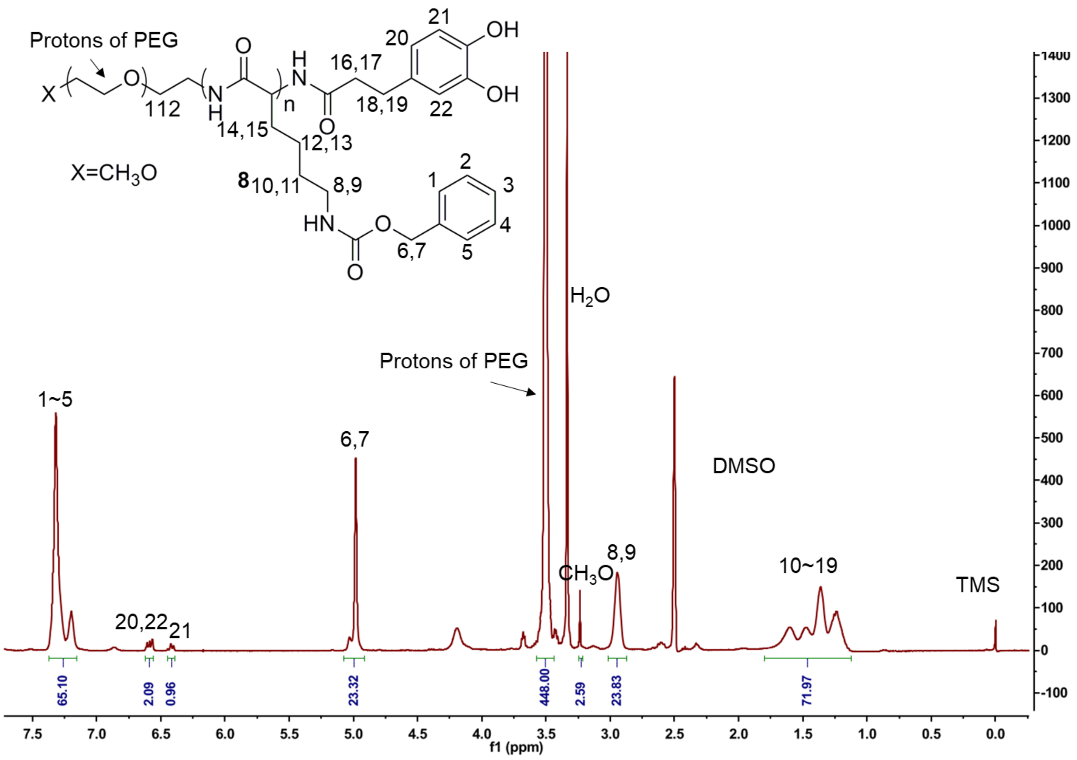

Figure S7. The ^{1}H NMR spectrum of **8** in DMSO-δ6.

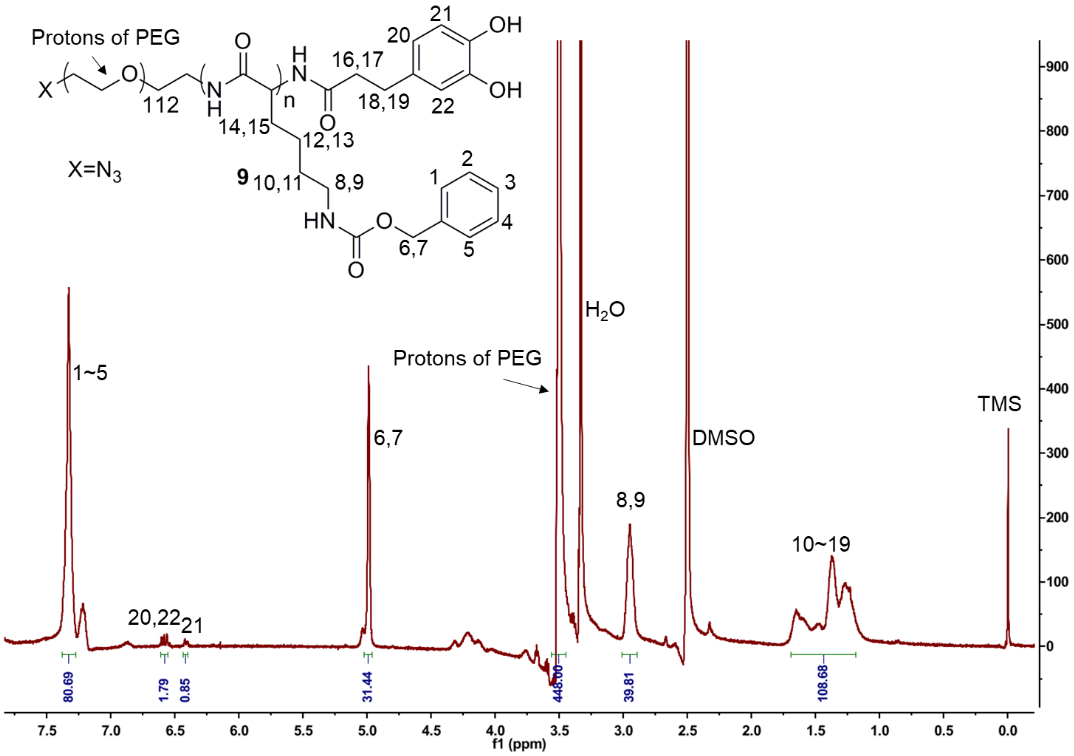

Figure S8. The ^{1}H NMR spectrum of **9** in DMSO-δ6.

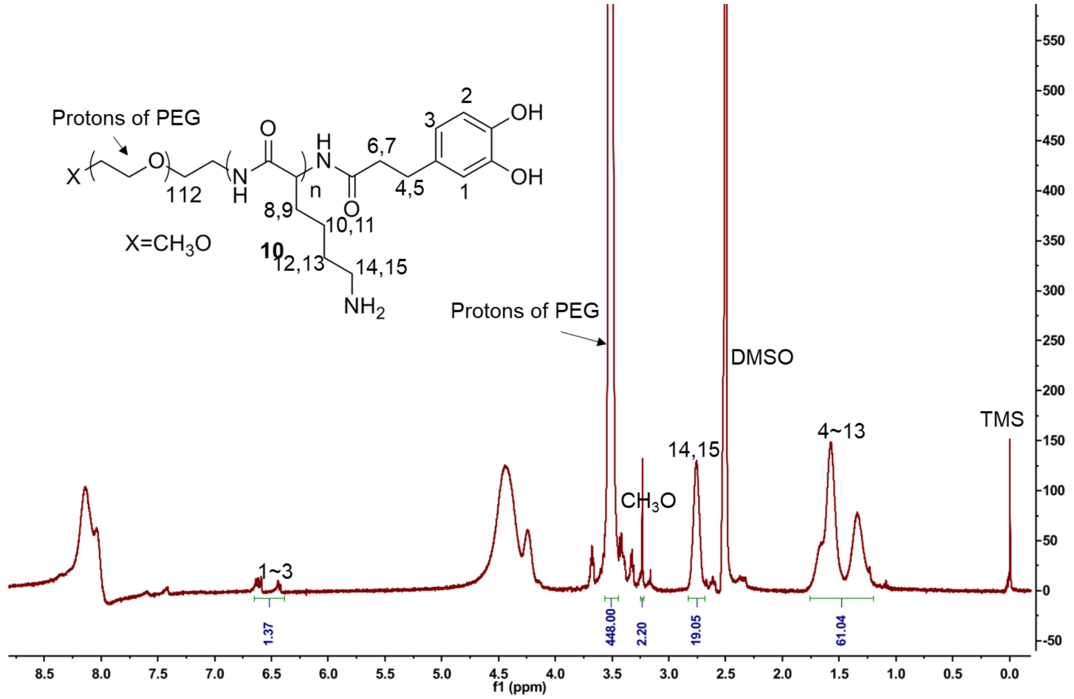

Figure S9. The ^{1}H NMR spectrum of **10** in DMSO-δ6.

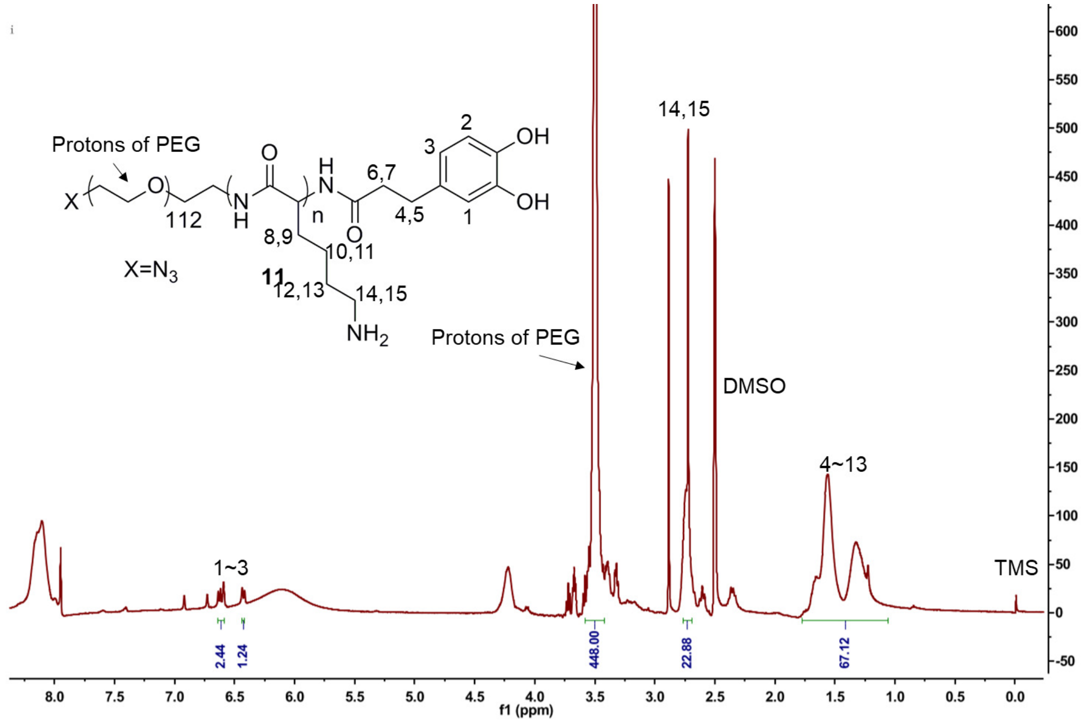

Figure S10. The ^{1}H NMR spectrum of **11** in DMSO-δ6.

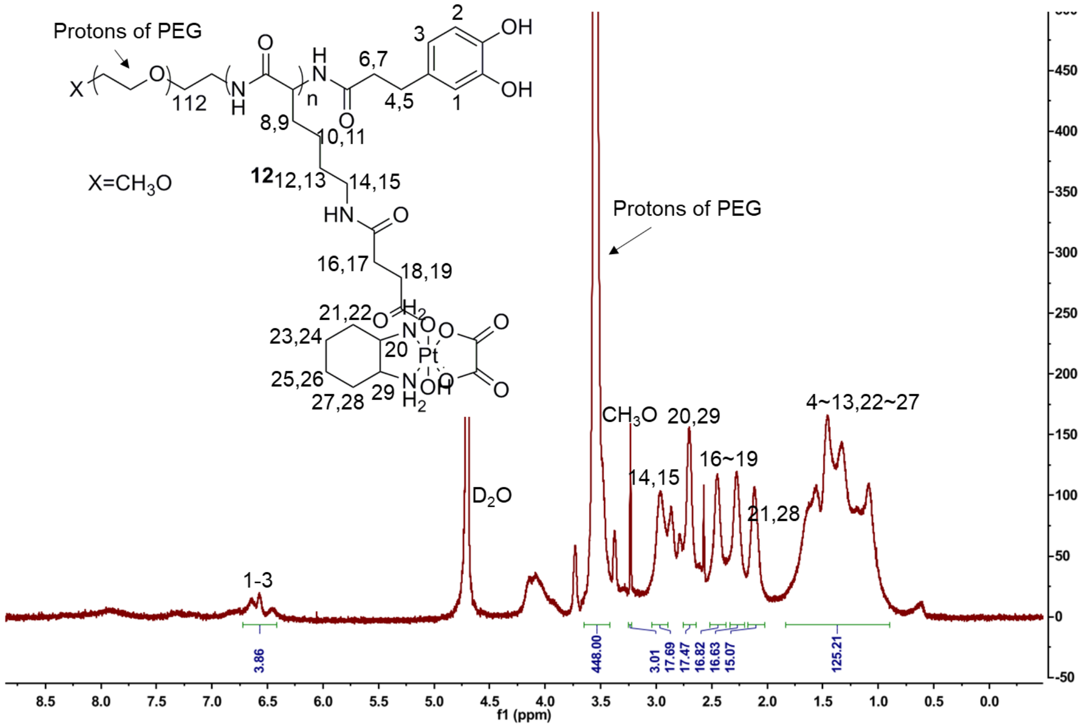

Figure S11. The ^{1}H NMR spectrum of **12** in DMSO-δ6.

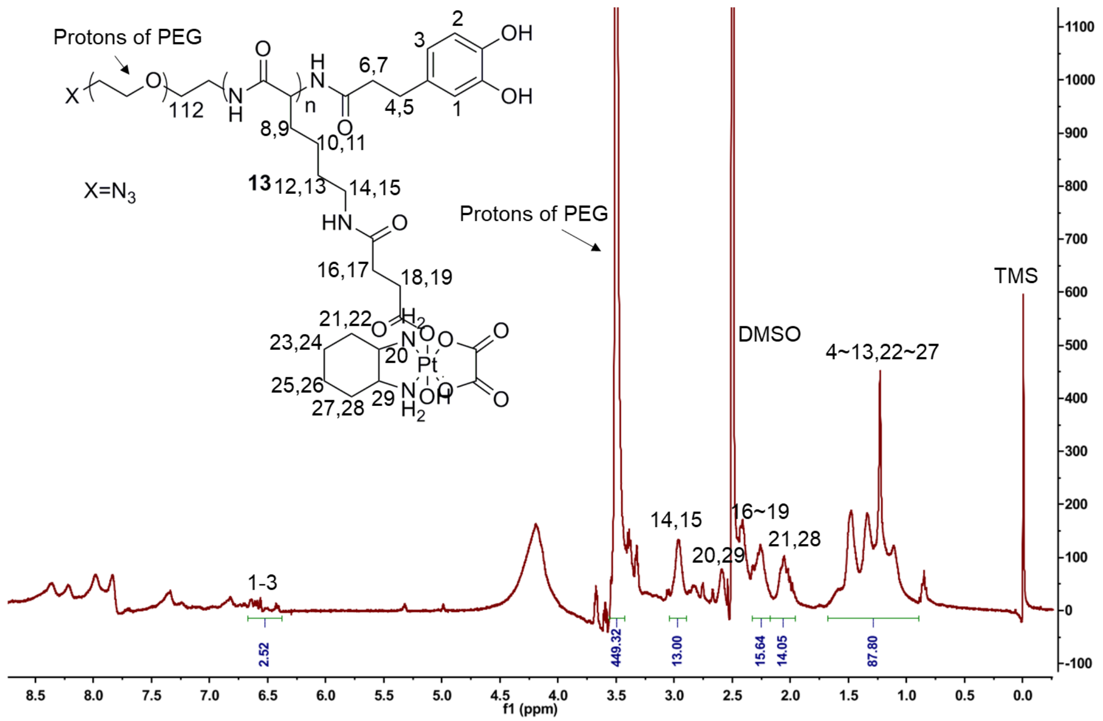

Figure S12. The ^{1}H NMR spectrum of **13** in DMSO-δ6.

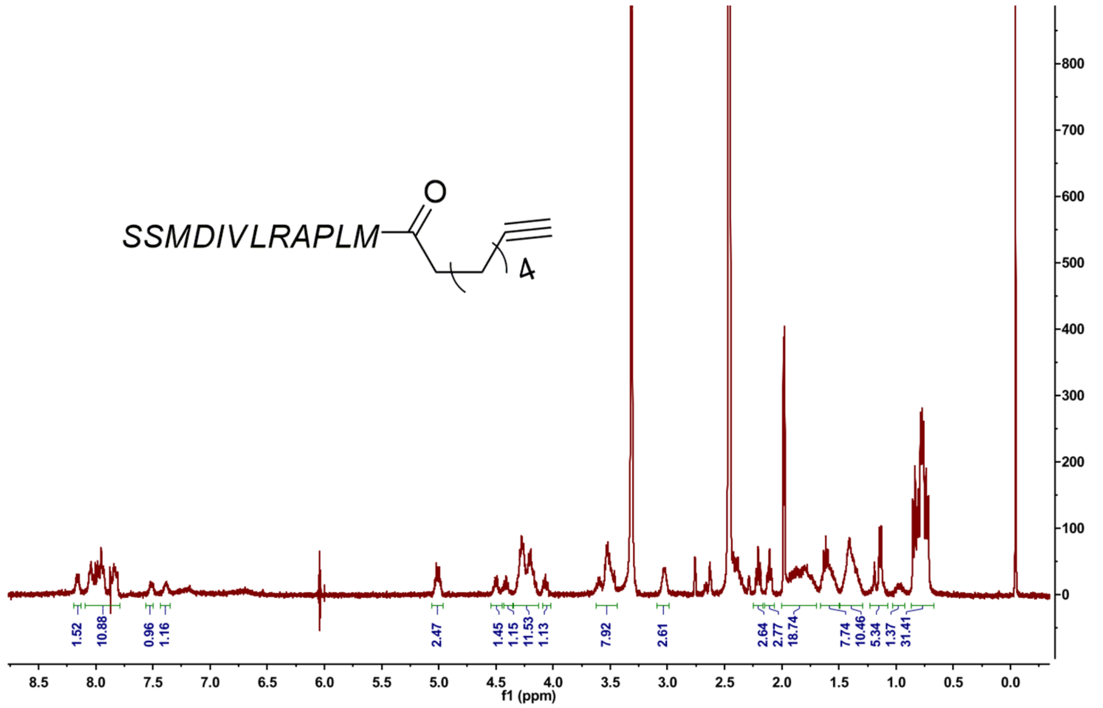

Figure S13. The ^{1}H NMR spectrum of **peptides** in DMSO-δ6.

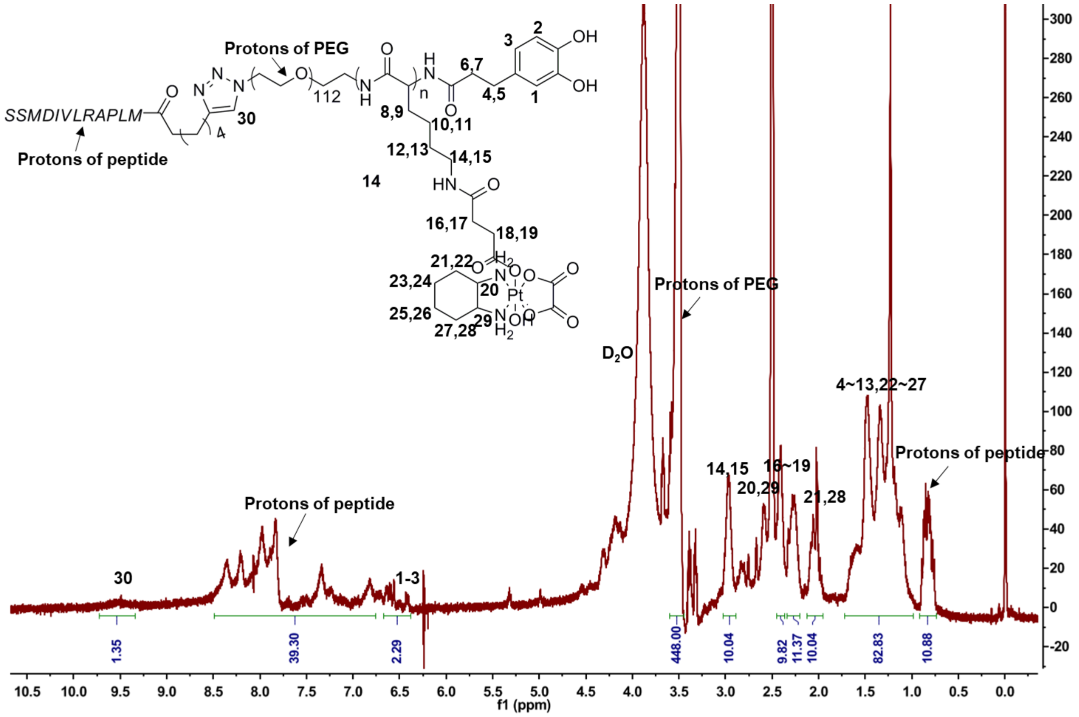

Figure S14. The ^{1}H NMR spectrum of **14** in D_2O.

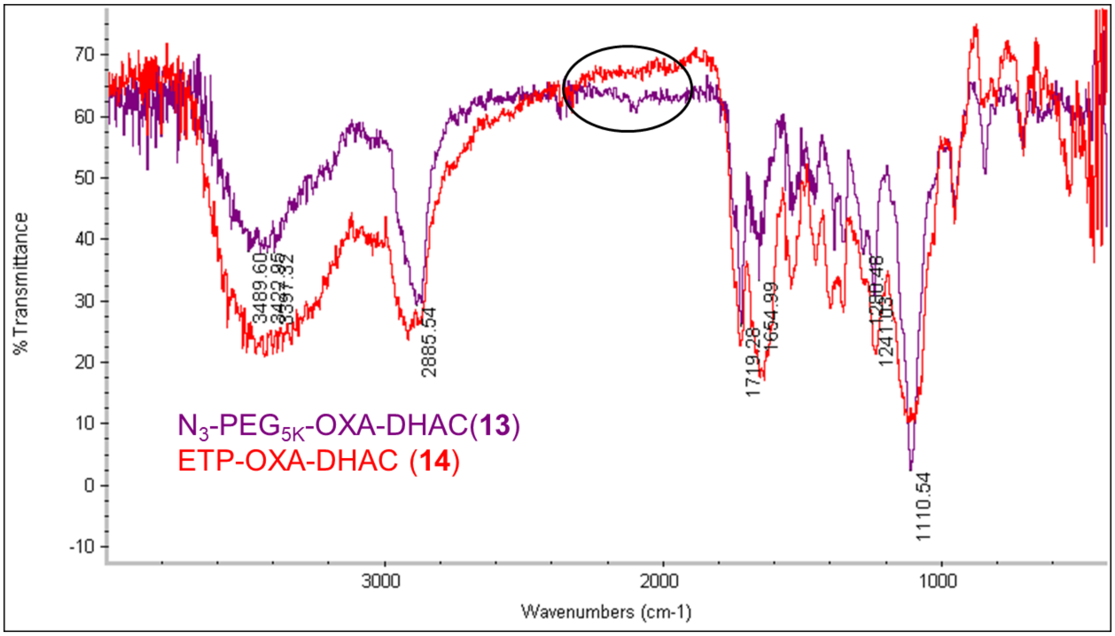

Figure S15. FT-IR spectrum of **13**,**14** in KBr tablet at ambient temperature. the circle indicates the disappearance of azide group at 2100 cm^{-1} after ETP peptide conjugation.

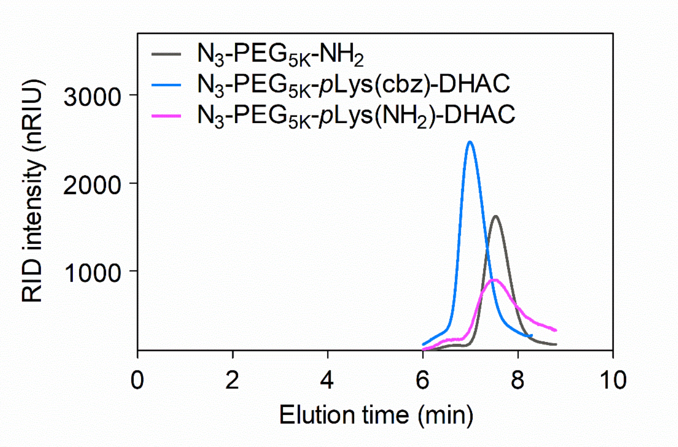

Figure S16. GPC elution times of N_3-PEG$_{5K}$-NH_2, N_3-PEG$_{5K}$-pLys(cbz)-DHAC (**7**) and N_3-PEG$_{5K}$-pLys(NH_2)-DHAC (**9**) were measured with a method: column: Phenogel™ 5μm 500 Å; flowing rate, DMF; detection by RID; flow rate=1 mL/min. The variation of elution time indicated the successful synthesis of compounds (**7, 8**).

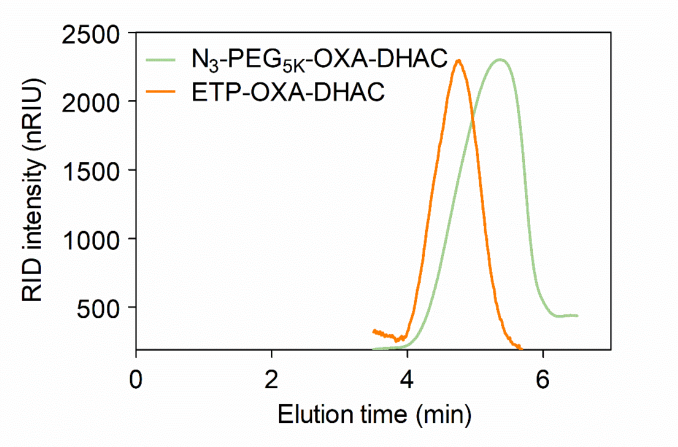

Figure S17. GPC elution time of N_3-PEG$_{5K}$-OXA-DHAC (**13**) and ETP-OXA-DHAC (**14**). The increase of elution time indicated the successful synthesis of compounds (**14**).

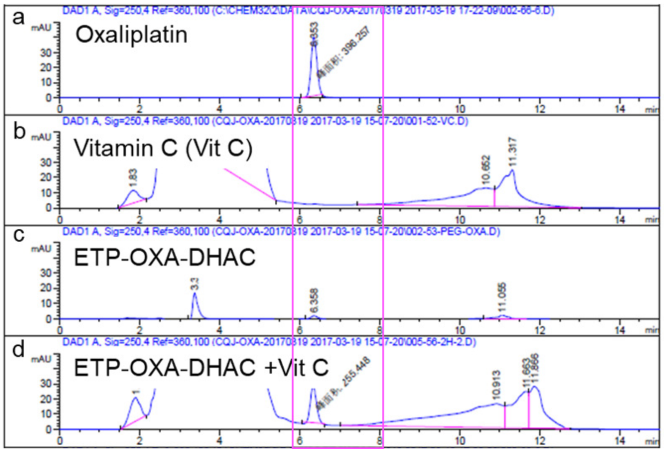

Figure S18. The HPLC spectra of oxaliplatin (a), Vitamin C (b), ETP-OXA-DHAC (c) and ETP-OXA-DHAC + Vit C (d). the increasing peak area at 6.35 min in **d** indicated the successful synthesis of compounds (**14**).

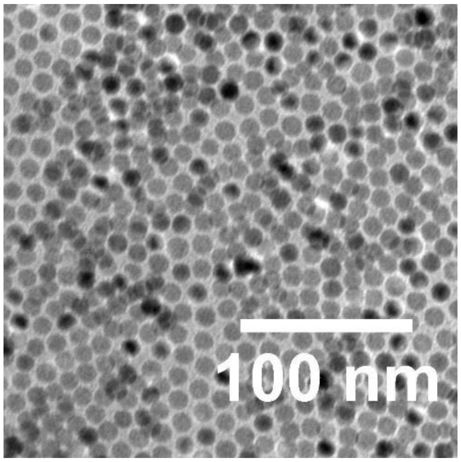

Figure S19. TEM images of oleic acid-Fe_3O_4. Scale bar, 100 nm.

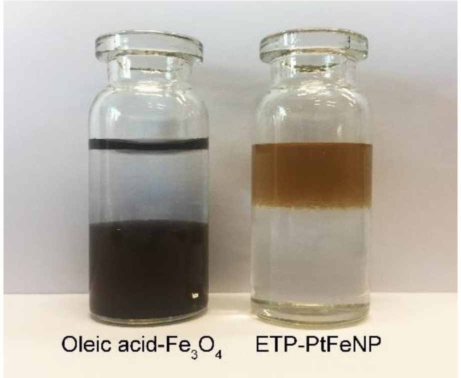

Figure S20. The digital photographs depict the hydrophilicity of Fe_3O_4 nanoparticles before (oleic acid-Fe_3O_4) and after the ETP-OXA-DHAC modification (ETP-PtFeNP). The top medium was water, while the bottom was dichloromethane.

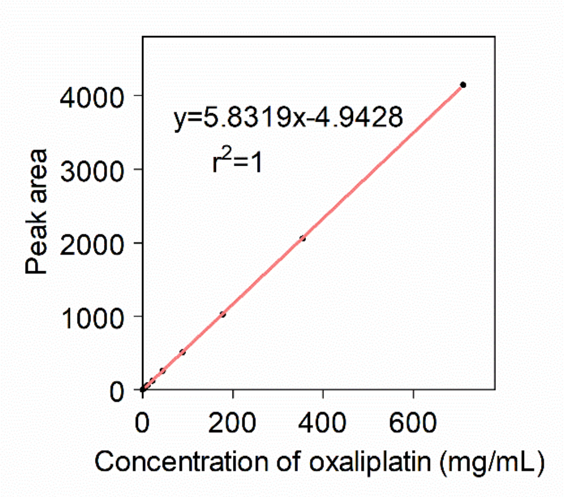

Figure S21. The standard curve of oxaliplatin by HPLC with a method: flowing rate, Methanol:H_2O=10:90 v:v; retention time of oxaliplatin, 6.2 min; detection by UV 250 nm; flow rate=1 mL/min; detection lower limit: 0.9 μg/mL.

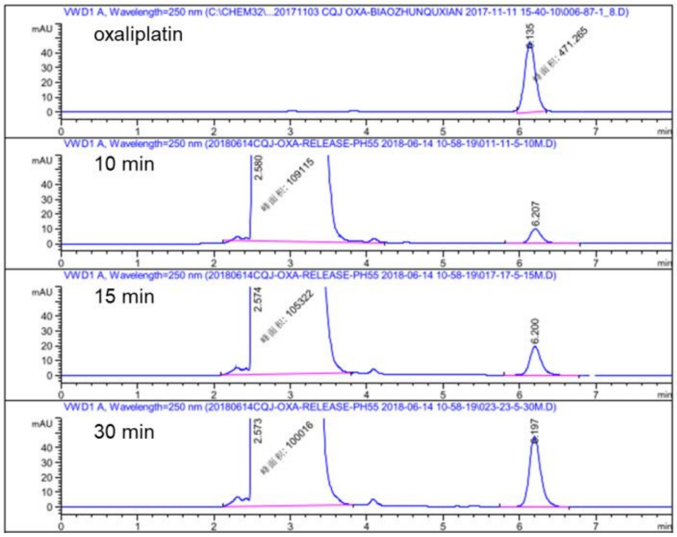

Figure S22. The HPLC spectra of oxaliplatin and supernatants of dissolution medium in which ETP-PtFeNP was incubated with PBS 5.5, 2 mM Vit C at different times.

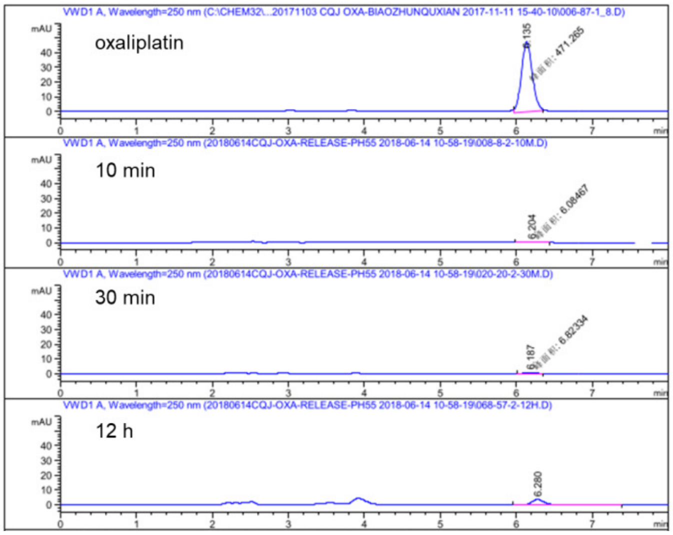

Figure S23. The HPLC spectra of oxaliplatin and supernatants of dissolution medium in which ETP-PtFeNP was incubated with PBS 5.5 at different times.

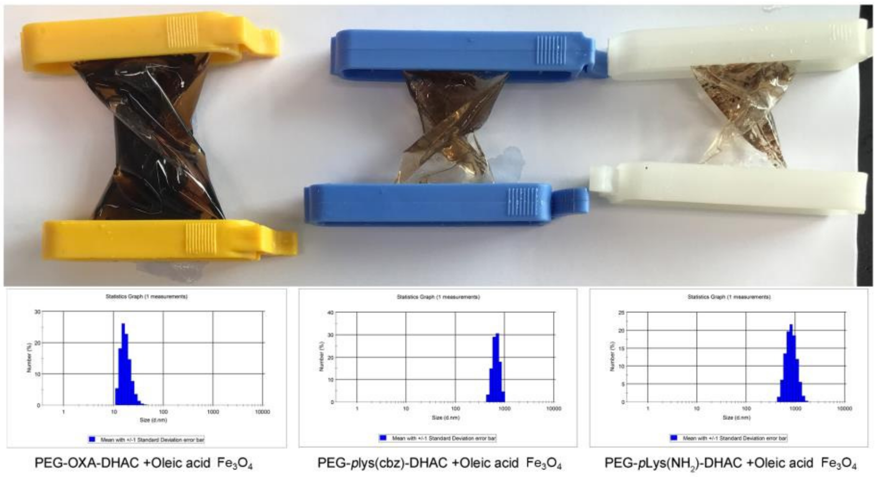

Figure S24. The photographs of the aqueous dialysis solutions (top) after the core-shell Fe3O4 nanoparticles was modified with PEG-OXA-DHAC, PEG-pLys(cbz)-DHAC and PEG-pLys(NH_2)-DHAC, respectively, and the corresponding DLS data of them (button).

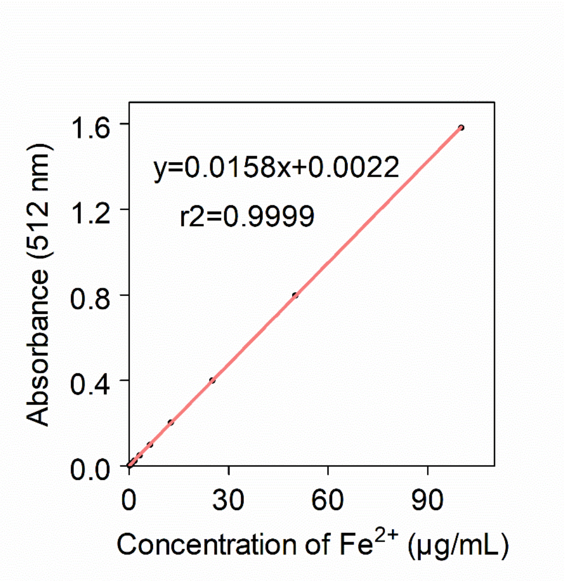

Figure S25. The standard curve of Fe^{2+} by Automatic microplate reader at UV 512 nm with the O-phenanthroline spectrophotometry.

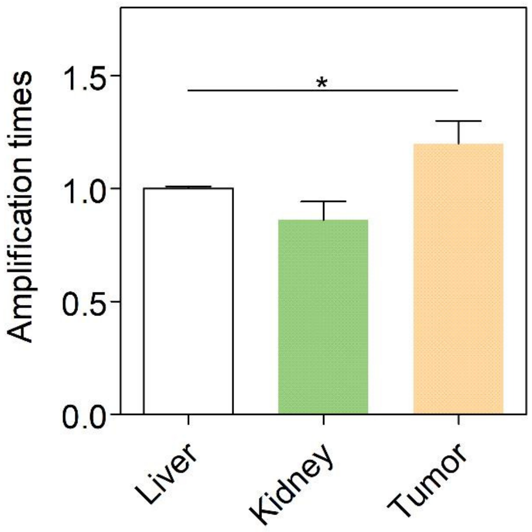

Figure S26. Q-PCR measuring the α-enolase mRNA in liver, kidney and 4T1 breast tumor tissue (n = 3). GAPDH: housekeeping gene.

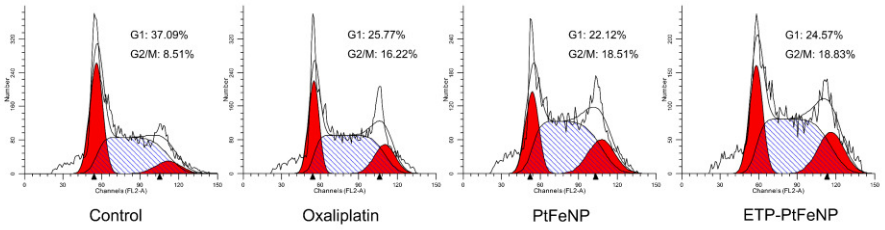

Figure S27. Cell cycle analysis by flowcytometry of 4T1 cells treated with different oxaliplatin formulations (oxaliplatin equiv. 15 μM).

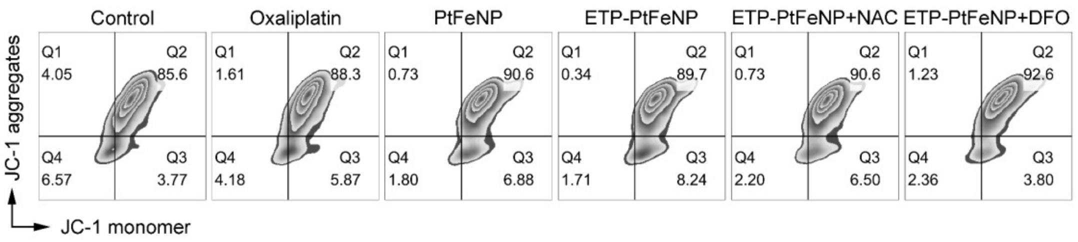

Figure S28. Increase in the mitochondrial membrane damage (the ratio of Q3 quadrant) in 4T1 cells on exposure to oxaliplatin (30 μM), PtFeNP and ETP-PtFeNP, from 3.77% (control) to 5.87%, 6.88% and 8.24%, respectively, for 12 h as assessed using JC-1 dye. The ratio of Q3 quadrant could be shifted down to 6.50% and 3.80% by co-incubation with NAC (5 mM) or DFO (100 μM), respectively.

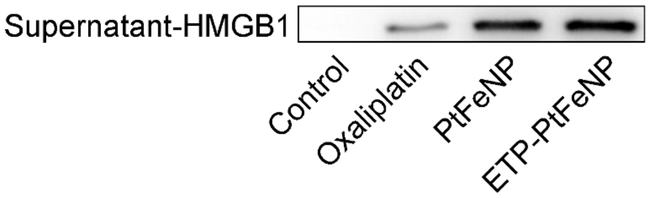

Figure S29. Western blotting analysis of HMGB1 release from the 4T1 cells into the extracellular fluid.

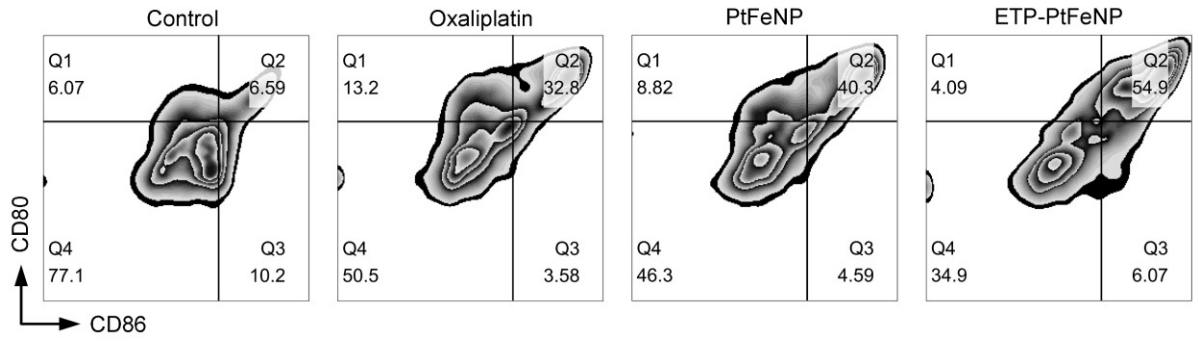

Figure S30. Flow cytometric analysis of DCs maturation in vitro.

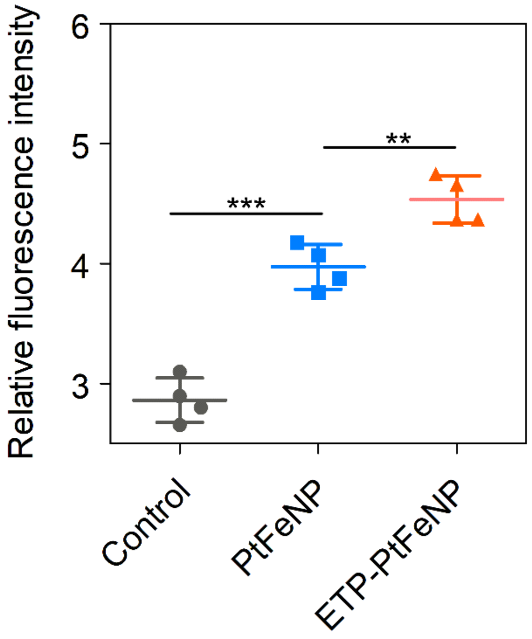

Figure S31. Semi-quantitative analysis of the Cy5.5-fluorescence of the tumor sections showing increased tumor-targeting accumulation of Cy5.5-labeled ETP-PtFeNP (n = 4). Error bar, mean ± s.d.

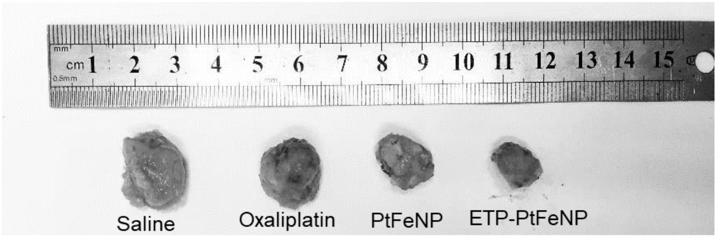

Figure S32. Representative tumor images at day 16 post-implantation.

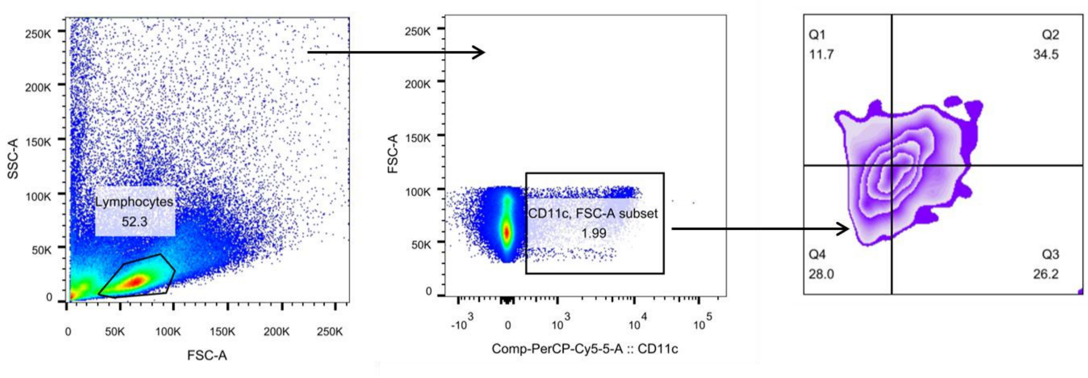

Figure S33. Gating strategy to determine frequencies of mature DCs from tumor draining lymph nodes.

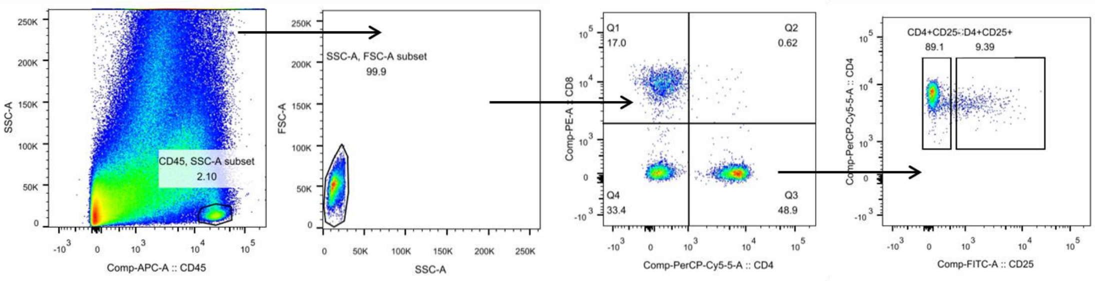

Figure S34. Gating strategy to determine frequencies of antitumoral immune cells from the tumor tissues.

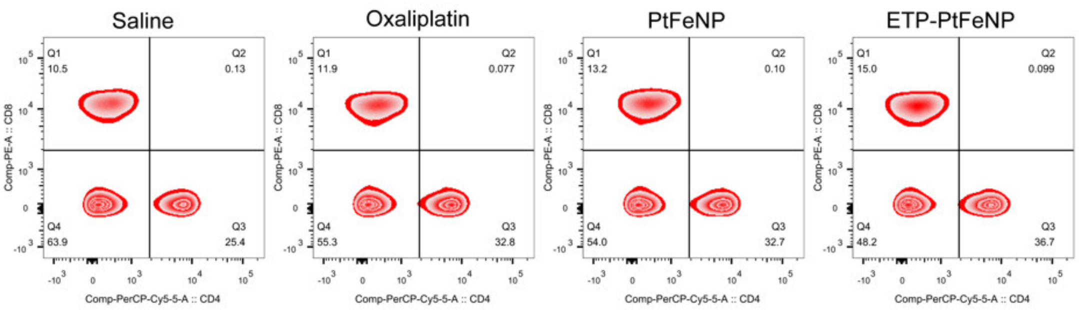

Figure S35. Representative plots of T cells in spleen of the 4T1 breast tumor-bearing mice after various treatments.

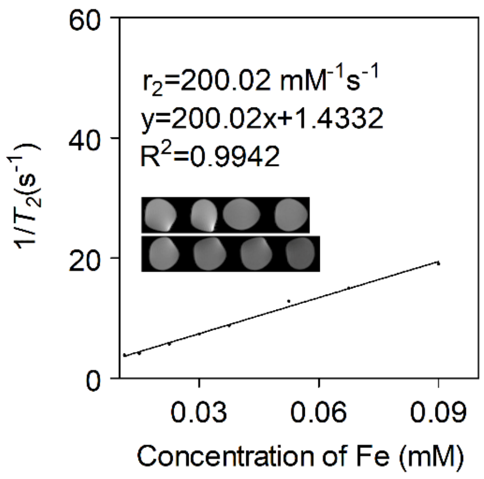

Figure S36. T_2 relaxation rate ($1/T_2$, black) as a function of Fe concentration in magnetic ETP-PtFeNP nanoparticles. The inset T_2-weighted MR images recorded for aqueous solutions of ETP-PtFeNP at various concentrations.

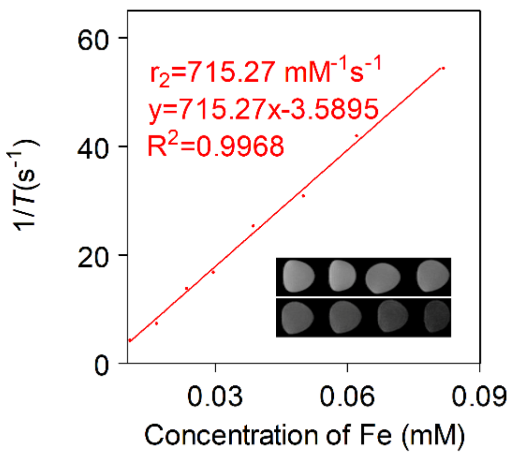

Figure S37. T_2 relaxation rate ($1/T_2$, red) as a function of Fe concentration in magnetic Vit C-processed ETP-PtFeNP nanoparticles. The inset T_2-weighted MR images recorded for aqueous solutions of Vit C-processed ETP-PtFeNP at various concentrations.

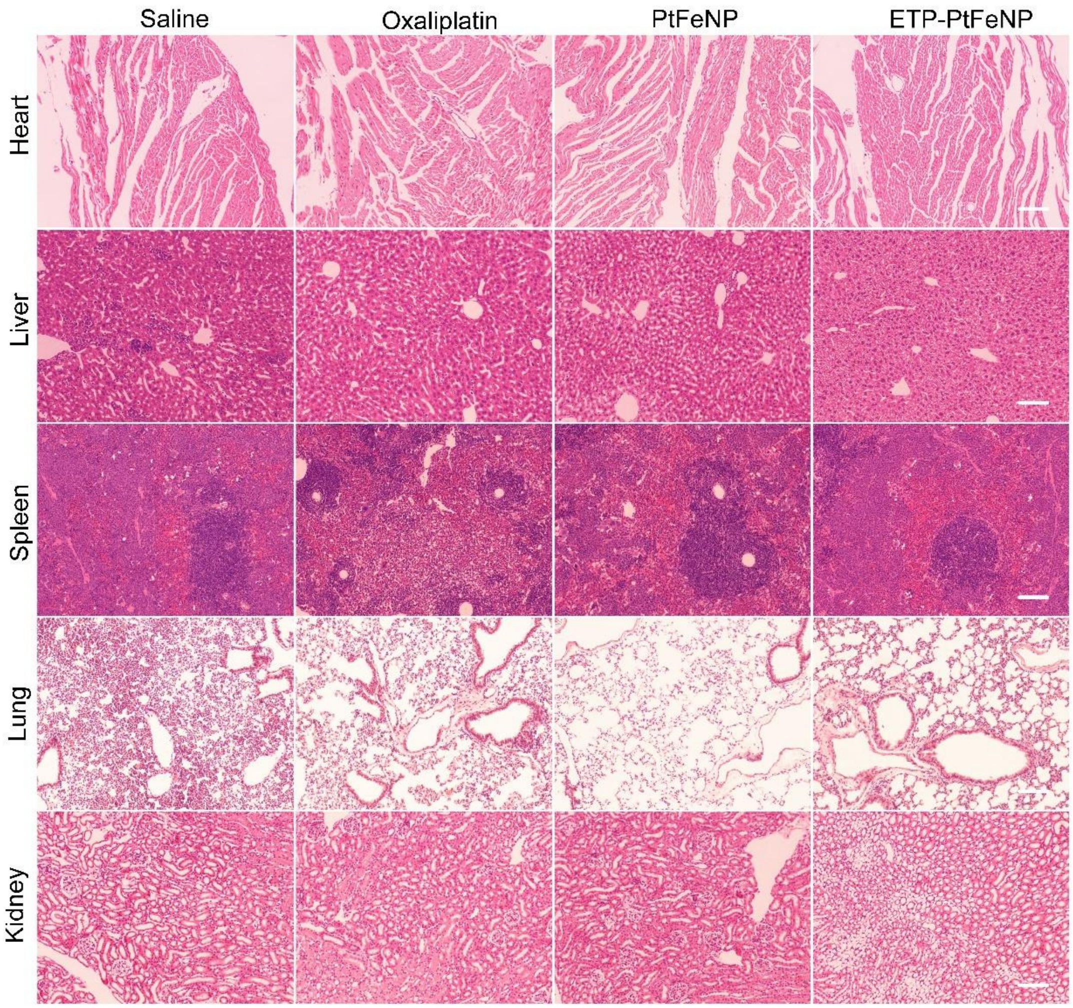

Figure S38. H&E staining of main organs from 4T1 breast tumor-bearing mice treated with different oxaliplatin formulations. Scale bars: 200 μm.

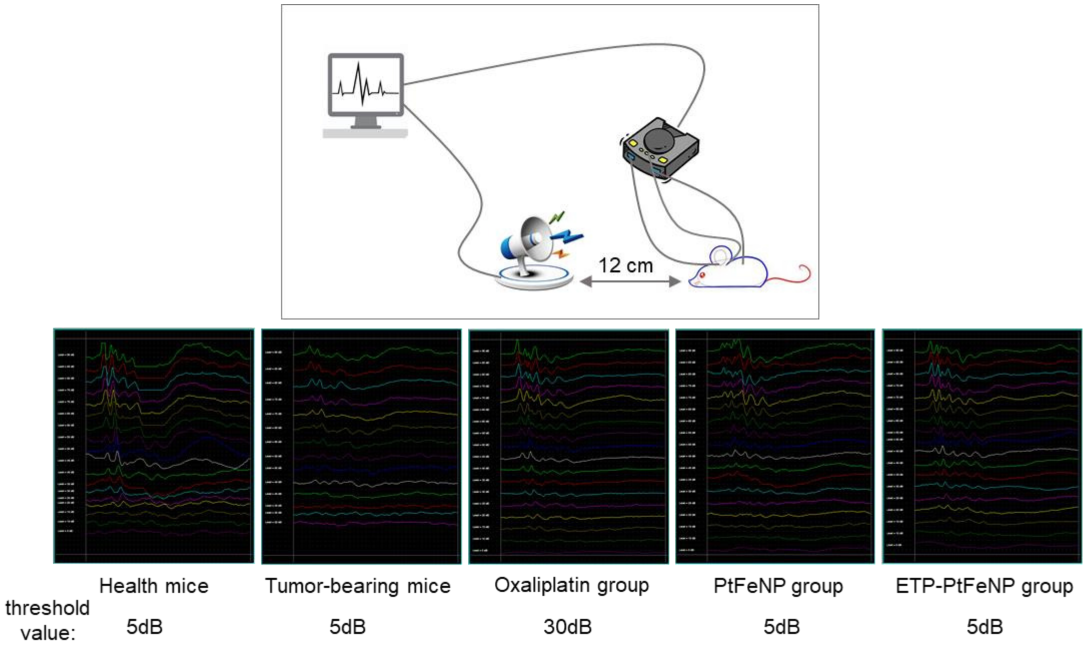

Figure S39. Schematic diagram of instruments for detecting audiometric thresholds for mice treated with different formulations, and the original neuroelectric signals over different sound intensity for healthy mice, where the thresholds can be determined upon the disappearance of the representative peaks.

Target	Host	Source and catalog number	application
Cytochrome C	Rabbit	Abcam, 133504	WB
Caspase-3	Rabbit	Abcam, 32351	WB
PD-L2	Rabbit	Abcam, 187662	WB, IF
Calreticulin	Rabbit	Abcam, 92516	WB, IF, Flow
HMGB1	Rabbit	Abcam, 79823	WB, IF
CD34	Rabbit	Abcam, 81289	IF
Rabbit IgG	Goat	Abcam, 150077	Alexa Fluor 488-conjugated second monoclonal antibody

CD45	Rat	eBioscience, 17-0451-82	Flow
CD8a	Rat	eBioscience, 12-0081-82	Flow
CD4	Rat	eBioscience, 45-0042-82	Flow
CD25	Rat	eBioscience, 53-0251-80	Flow
CD11c	Armenian hamster	eBioscience, 45-0114-80	Flow
CD80	Armenian hamster	eBioscience, 17-0801-82	Flow
CD86	Rat	eBioscience, 12-0862-82	Flow

Table S1. Antibody information.

Experimental section

Materials. α-Methoxy-ω-amino-poly (ethylene glycol) (CH_3O-PEG-NH_2, Mw 5,000) was purchased from Seebio Biotech (Shanghai, China). α-Azide-ω-amino-poly (ethylene glycol) (N_3-PEG-NH_2, Mw 5,000) was purchased from Jenkem Technology (Beijing, China). α-Enolase targeting peptide (sequence: SSMDIVLRAPLM-(5-Hexynoic acid)) was synthesized by ChinaPeptides Co., Ltd. (Nanjing, China). Oxaliplatin was purchased from Meilunbio (Dalian, China). Oleic acid-Fe_3O_4 nanoparticles were a kind gift from Prof. Zhijie Yang from Shandong University. 3-(4,5-dimethyl-2-thiazolyl)-2,5-diphenyl-2H tetrazolium bromide (MTT), 2′,7′-dichlorofluorescein diacetate (H_2DCFH-DA) and 4',6-diamidino-2-phenylindole (DAPI) were purchased from Sigma-Aldrich (St. Louis, USA). Hoechst 33342 and LysoTracker DND Green were purchased from Molecular Probes (Waltham, USA). Cy5.5-NHS (684/710 nm) was purchased from APExBIO (Houston, USA). Terminal deoxynucleotidyl transferase dUTP nick-end labeling (TUNEL) assay and annexin V-FITC/PI apoptosis detection kit were purchased from KeyGEN BioTECH (Nanjing, China). Mitochondrial membrane potential assay kit (JC-1) and ER-Tracker Red were purchased from Beyotime (Shanghai, China). IFN-γ ELISA kit was purchased

from Neobiosconece (Shenzhen, China). All the other reagents were purchased from Energy Chemical (Shanghai, China).

Cell lines. 4T1 cells were cultured in RPMI 1640 medium supplemented with 10% fetal bovine serum, 10 U/ml penicillin and 10 μg/ml streptomycin at 37 °C and 5% CO^2 in a humidified atmosphere.

Mouse model establishment. Female BALB/c mice of ~20 g body weight were obtained from Department of Experimental Animals (Fudan University) and maintained under standard laboratory conditions. All animal handling procedures were approved by Institutional Animal Care and Use Committee of China. Orthotopic 4T1 breast tumor model was established by injection of 1.0×10^6 4T1 cells in 100 μL PBS 7.4 into the second right breast pad of female BALB/c mice.

Preparation and characterization of prodrug-loaded formulations. ETP-PtFeNP and PtFeNP nanoparticles were prepared via phase inversion dialysis method. In detail, PEG-OXA-DHAC and ETP-OXA-DHAC polymers were dissolved in anhydrous N,N-Dimethylformamide (DMF) as stock solution A (0.001 mmol/mL), and the oleic acid-Fe_3O_4 nanoparticles were dissolved in anhydrous tetrahydrofuran to form stock solution B (0.004 mmol/mL). Stock solution A (1 eq.) was added into stock solution B (3 eq.) under an ultrasonic dispersion, which was then transferred to turbine mixer for further mixing. As for Cy 5.5-labeled nanoparticle's preparation, the Cy5.5-NHS was added into the mixing solutions, which could attach to the free amino group of polymers via amidation reaction. After vortex mixing for 3 h, the solution sealed in a dialysis bag (diameter = 40 mm, MWCO = 20,000) followed by dialysis against DMF for 48 h, and then dialyzed against deionized water for another 48 h. A transparent solution was collected and filtered through a 0.22 μm filter, then freeze-dried to give ETP-PtFeNP and PtFeNP as a brown floccule. Sizes of the freshly prepared nanoparticles were measured in deionized water by dynamic light

scattering (DLS) (Zetasizer Nano-ZS, Malvern, U.K.). The morphological images of the formulations were photographed by transmission electron microscope (TEM, Tecnai G2 spirit Biotwin, FEI). Furthermore, the stability test of nanoparticles was carried out in PBS 7.4 for 7-day incubation, and the size and zeta potential of the nanoparticle were measured by DLS.

In-vitro drug release study. To study the redox-sensitive release property of formulations, ETP-PtFeNP nanoparticles were incubated with PBS 7.4, 2 mM Vit C, and the size distribution and morphology of nanoparticles were measured by DLS and TEM, respectively. In-vitro prototype platinum drug release from ETP-PtFeNP was performed using dialysis method (n=3). In brief, 500 μL ETP-PtFeNP solution (16 mg/mL) in dialysis bag (MWCO = 3,500 Da) was immersed in 10 mL dialysis medium (PBS 7.4 or PBS 5.5 or PBS 7.4, 10 mM Vit C or PBS 5.5, containing 2 mM or 10 mM Vit C) and the experiment was operated in a shaking bath at 100 rpm, 37 °C. At the predetermined times, the external medium was collected and the equal volume of fresh dialysis medium was supplemented. Oxaliplatin contents in these samples were detected by HPLC measured by UV detector at 250 nm (10% CH_3OH, 90% H_2O).

In-vitro Ferric ions release study. Ferric ions release from ETP-PtFeNP nanoparticles was performed using dialysis method (n=3). In detail, 500 μL ETP-PtFeNP solution (16 mg/mL) in dialysis bag (MWCO = 3,500 Da) was immersed in 10 mL dialysis medium (PBS 7.4 or PBS 5.5 or PBS 5.5, 2 mM Vit C) and the experiment was operated in a shaking bath at 100 rpm, 37 °C. At the predetermined times, the external medium was collected and the equal volume of fresh dialysis medium was supplemented. The Ferric ions contents in these samples were measured by O-phenanthroline spectrophotometry.

Quantitative real-time PCR (q-PCR) assay. The expressions of α-enolase at the mRNA level in excised 4T1 breast tumor tissues, liver and kidney were examined by semi-quantitative q-PCR.

Total RNA was extracted using a TRIzol reagent and subjected to the synthesis of first-strand cDNA using a reverse transcription kit. The specific primers were synthesized as follows: ENO-1-sense: 5'-CGTACCGCTTCCTTAGA AC-30; ENO-1-antisense: 50-GATGACACGAGGCTCACA-30; GAPDH-sense: 5'-ACCCACTCCTCCACCTTTG-3'; GAPDH-antisense: 5'-CTCTTGTGCTCTTGCTGGG-3'.

Cell uptake study. 4T1 cells were seeded in a 24-well plate (Corning-Coaster, Tokyo, Japan) at a density of 3×10^4 cells/well. When achieving 80–90% confluence, the cells were incubated with Cy5.5-labeled PtFeNP and ETP-PtFeNP with an equal concentration of Cy5.5 (1 μg/mL) in serum-free RPMI for 60 min. Then the cells were rinsed with Hank's for three times. The cellular uptake of nanoparticles was visualized and photographed by Confocal Laser Scanning Microscope (CLSM, Carl Zeiss LSM710, Carl Zeiss, Jena, Germany) and quantified by flow cytometer (FACS, BD Biosciences, Bedford, MA). The cellular internalization mechanism of the ETP-PtFeNP was also investigated using ETP peptide as an inhibitor.

Intracellular distribution of ETP-PtFeNP. To investigate the intracellular distribution of ETP-PtFeNP, we performed immunofluorescence study to visualize the lysosome in cells treated with ETP-PtFeNP. 4T1 cells were seeded in 35-mm confocal dishes (5×10^3 cells/well). After 12-h incubation, cells were treated with Cy5.5-labeled ETP-PtFeNP for 15 min or 1 h, and then the cells were rinsed with Hank's for three times. Afterwards, the cells were stained with LysoTracker™ Green DND-26 to visualize the lysosome, and the cell nuclei were stained with Hoechst 33342. The intracellular distribution of ETP-PtFeNP was visualized and photographed by CLSM.

Cytotoxicity assay. MTT assay was used to evaluate the cytotoxicity of free oxaliplatin, PtFeNP and ETP-PtFeNP. Briefly, 4T1 cells were seeded in 96-well plates at a density of 5,000 cells per well and cultured in 5 % CO^2 at 37 °C for 12 h. Then 200 μL of oxaliplatin, PtFeNP or ETP-

PtFeNP dissolved in the culture medium was added to the 96-well plates. The final concentration range for each group was set as 1 nM to 400 μM Pt-based concentration. After 48-h incubation, the culture media was removed and the residuals in the wells were washed three times with PBS 7.4. Then, 200 μL of 0.5 mg mL–1 MTT solution in PBS 7.4 was added, and the plate was incubated for another 4 h at 37 °C. After that, the supernatant was removed and 200 μL of DMSO was added to each well to dissolve the formazan crystals. Finally, the plates were shaken for 10 min at dark, and the absorbance of the formazan product was measured at 570 nm by using a Multiskan MK3 microplate reader (Thermo Scientific, Waltham, Massachusetts, USA). Cell viability was calculated as the survival percentage of control.

Cell cycle analysis. 4T1 cells were seeded on a six-well plate with a density of 2×10^5 cell per well and cultured at 37 °C for 12 h. When reaching 50–60% confluence, cells were incubated in FBS-free medium for another 12 h. Then cells in each well were treated with oxaliplatin, PtFeNP and ETP-PtFeNP with an equal concentration (15 μM Pt-based concentration) in FBS-free medium for 24 h. Population of 4T1 cells in different stages of the cell cycle was stained with PI under the instructions of DNA content Quantitation Assay and quantified by flow cytometer. Population and histograms of cell number versus PI intensity were used to determine the percentage of cells in each phase of the cell cycle.

Measurement of ROS generation. To determine the formation of ROS, H_2DCFH-DA, which could be oxidized to the highly fluorescent dichlorofluorescein (DCF) by ROS, was chosen to detect the intracellular generation of ROS. Briefly, 4T1 cells were seeded into 96-well plates with a density of 5×10^4 cells per well and cultured at 37 °C for 12 h. Cells were treated with oxaliplatin, PtFeNP, ETP-PtFeNP at an equal Pt concentration of 90 μM and Fe concentration of 156 μM for 2 h. To block the iron-mediated ROS generation which induces the intercellular ROS generation,

100 μM iron chelator deferoxamine mesylate (DFO) and 5 mM ROS scavenger, N-acetyl-L-cysteine (NAC) was used as a control, respectively. After rinsed with Hank's for three times, 100 μL H_2DCFH-DA were added at concentration of 20 μM and the cells were cultured in 5% CO_2 for 2 h. Afterwards, the fluorescence intensity was measured by Multiskan MK3 microplate reader at λex/em, 485 nm/ 528 nm.

Visualization of ROS production in the ER by confocal microscopy. 4T1 cells were plated at 5,000 cells/well in 35-mm confocal dishes in heat deactivated complete RPMI 1640 medium. Cells were treated with ETP-PtFeNP at Pt concentration of 90 μM for 4 h before washed thrice with Hank's and incubated with 20 μM pre-warmed H_2DCFH-DA (488 /528 nm) for 20 min at 37 °C. After 20 min, H_2DCFH-DA was removed. 1 μM pre-warmed ER-Tracker Red (587 /615 nm) was added to the cells, and cultured for another 20 min. Afterwards, the cells were rinsed with Hank's for three times and then analyzed with CLSM.

Mitochondrial membrane potential assay. Mitochondrial membrane potential (MMP) was measured by detecting a shift from red (485 /590 nm) to green (485 /525 nm) fluorescence while using JC-1 as a sensor. Briefly, 4T1 cells (2×10^5) were seeded in 6-well plates and cultured for 12 h under 5% CO_2 at 37 °C. Then the cells were treated with fresh medium containing oxaliplatin, PtFeNP, and ETP-PtFeNP, respectively, at an equal Pt concentration of 30 μM for 12 h, and co-treatment with NAC (5 mM) or DFO (100 μM) as inhibitors. After incubation, the medium was removed and the cells were washed twice with cold PBS 7.4 and the cells were then stained with JC-1 (5 mg/mL) at 37 °C for 30 min at dark. Stained cells were washed twice with cold JC-1 buffer solution to remove free dye and were observed by CLSM. The MMP of the 4T1 cells in each treatment group were calculated as the ratios of red to green fluorescence gained from the CLSM

image by ImageJ (ImageJ 1.x, National Institutes of Health, America). Meanwhile, the stained cells also were quantified by flow cytometry.

Western blot. Immunoblotting analysis was carried out as previously described. For abbreviation, 4T1 cells treated with different formulations (at an equal Pt concentration of 30 μM for 12 h) were lysed with RIPA lysis buffer. Then, cell lysates and purified membrane vesicles samples were resolved on 14 % SDS-PAGE and analyzed by immunoblotting using Cytochrome C (Cyt C), cleaved caspase-3 and β-actin antibodies, followed by enhanced chemiluminescence (ECL) detection (Millipore, Darmstadt, Germany). To investigate the protein expression of Cyt C, cleaved caspase-3, CRT, HMGB1 and PD-L2 at the tumor sites, the tumor tissues were isolated from treated mice, homogenated and lysed with RIPA lysis buffer. Then, cell lysates and purified membrane vesicles samples were resolved on 14 % (for analysis of Cyt C and cleaved caspase-3) or 10 % (for analysis of CRT, HMGB1 and PD-L2) SDS-PAGE and analyzed by immunoblotting using corresponding antibodies, followed by ECL detection.

Apoptosis assay. Apoptosis in 4T1 cells was measured by using the Annexin V-FITC/ PI staining kit. The cells were seeded in a six-well plate with a density of 4×10^5 cells/well for 12 h. And cells were treated with free oxaliplatin, PtFeNP and ETP-PtFeNP with a final oxaliplatin concentration of 30 μM for 12 h. Co-treatment with NAC (5 mM) or DFO (100 μM) as inhibitors. After exposure, cells were trypsinized and centrifuged at 2,000 rpm for 5 min, washed with cold PBS 7.4 three times, re-suspended in binding buffer and stained with Annexin V-FITC/PI according to the instructions at room temperature in darkness for 15 min. then the apoptotic cells were evaluated by gating PI and Annexin V-positive cells on flow cytometry.

Flow cytometric analysis of CRT on the cell surface. 4T1 cells were seeded into 6-well plates at a density of 2×10^5 cells/well and cultured at 37 °C for 12 h. After treated with the indicated

agents for 4 h, the cells were collected and washed thrice with cold Hank's. Then the cells were incubated with CRT primary antibody (diluted in cold blocking buffer which contains 2% fetal bovine serum) for 1 h, followed by washing and incubation with the appropriate Alexa 488-conjugated monoclonal secondary antibody in blocking buffer (for 1 h). Each sample was analyzed by flow cytometry to identify cell surface CRT. Antibodies used in flow cytometry are listed in Supplementary Table 1.

HMGB1 release assay. 4T1 cells were plated in 6-well plates at a density of 2×10^5 cells per well. After 24 h treatment with different formulations (at an equal Pt concentration of 90 μM), the supernatants were collected, dying cells were removed by centrifugation and supernatants were concentrated to 100 μL by ultrafiltration. Released HMGB1 in the supernatant and HMGB1 was detected via western blot.

Immunofluorescence. For surface detection of CRT, the cells were seeded in 35-mm confocal dishes at a density of 1×10^5 cells /well for 12 h. After treated with the indicated agents for 4 h, the cells were washed thrice with cold Hank's and fixed in 0.1% paraformaldehyde in PBS for 5 min at 0 °C. Then the cells were washed thrice in cold Hank's, and CRT primary antibody (diluted in cold blocking buffer) was added for 1 h. After three washes in cold Hank's, the cells were incubated with the Alexa 488-conjugated secondary antibody for another 1h. The cells were fixed with 4% paraformaldehyde for 10 min before nuclear staining with DAPI (1/1,000) in cold PBS. For intracellular HMGB1 staining, the cells were treated with the indicated agents for 4 h after seeded in 35-mm confocal dishes at a density of 105 cells per well for 12 h. The cells were rinsed three times with cold Hank's, fixed in 4% paraformaldehyde for 10 min, permeabilized with 0.1% Triton X-100 for 10 min and washed thrice with cold Hank's. After blocked with 10% fetal bovine serum in Hank's for 30 min, HMGB1 primary antibody was added for 1 h. Subsequently, the cells

were washed three times with cold Hank's, incubated with an Alexa Fluor 488-conjugated secondary antibody for 1 h and stained with DAPI (1/1,000) in cold PBS. For section staining, first, to remove the embedding medium, the frozen tumor sections were washed with PBS 7.4 for 10 min×3. The sections were incubated at 0 °C overnight with a primary antibody, diluted in blocking buffer (2% fetal bovine serum and 0.5% Triton X-100), and then rinsed three times with PBS. Afterwards, the specimens were incubated with a fluorescence-labeled secondary antibody for 2 h in the dark. Before detection by CLSM, the nucleus was stained with DAPI and then washed thrice with PBS for10 min ×3. Antibodies used in immunofluorescence are listed in Supplementary Table 1.

DCs maturation in vitro. Bone marrow-derived DCs (BmDCs) were prepared as described previously.[1] Briefly, BmDCs was collected from tibias and femurs of female BALB/c mice, passed through a 70 μm cell strainer to remove small pieces of muscle and bone, resuspended in RPMI 1640 medium, containing 15% FBS for 4 h. Then, the adherent cells were cultured for 5 days in RPMI 1640 medium, containing 15% FBS, 10 ng/mL GM-CSF (Sigma) and 10 ng/mL IL-4 (Sigma). On day 5, DCs were seeded into 24-well plates at a density of 2×10^5 cells/well, and the medium was replaced by conditioned medium of 4T1 cells treated with different formulations (at an equal Pt concentration of 90 μM) for 24 h. after 24 h, the mature DCs were analyzed via flow cytometric following staining. Fluorescence conjugated antibodies used in flow cytometry are listed in Supplementary Table 1.

In-vivo imaging and biodistribution studies. Cy5.5-labeled ETP-PtFeNP or PtFeNP nanoparticles were injected intravenously via tail vein into 4T1 breast tumor cells-bearing mice at a dose of 0.1 mg Cy5.5 (684/710 nm) /kg. The in-vivo biodistribution of nanoparticles at various time points was traced and visualized by Xenogen IVIS Spectrum CT (Perkin Elmer Inc., Waltham,

Massachusetts, USA). While at 24 h after injection, mice were sacrificed. The ex-vivo biodistribution of Cy5.5-labeled nanoparticles was also assessed and quantified by IVIS.

Intra-tumoral distribution study. After injected with Cy5.5-labeled ETP-PtFeNP or PtFeNP nanoparticles for 24 h, the mice were sacrificed. The frozen tumor slices were stained with CD34 primary antibody and an Alexa Fluor 488-conjugated secondary antibody to visualize the blood vessels, and the nuclei were stained with DAPI. The fluorescence was observed under confocal microscope. antibodies used in immunofluorescence are listed in Supplementary Table 1.

T_2-weighted MRI study. For magnetization curves, magnetization measurements were performed on a Magnetic Property Measurement System 3 (MPMS3, Quantum Design). The experiments were performed in a 7 T MR scanner for small animal imaging system (BioSpec70/20USR, Burke, Germany). For in-vitro T_2-weighted MRI studies, ETP-PtFeNP or Vit C-processed samples were dispersed in water at various Fe concentrations (by O-phenanthroline spectrophotometry). The transverse relaxation time (T_2) was acquired by using a spin-echo pulse sequence for investigating the MR signal decreasing effects. Subsequently, the T_2-weight relaxivity values r_2 were determined through the curve fitting of $1/T_2$ relaxation time (s^{-1}) versus the Fe concentration (mM). For in-vivo T_2-weighted MRI studies, the 4T1 breast tumor-bearing Balb/c mice at a tumor size 100 mm^3 were intravenously injected with ETP-PtFeNP or PtFeNP at a Fe dose of 5 mg/kg body weight. Thereafter, T_2-weitghted MR images across the tumor in transverse plane were acquired before and at different time points of 30, 60, 120, 180 min post administration, following by using Rapid Acquisition Relaxation Enhanced (RARE) sequence (Respiratory gating contained) under the parameters: scan = 13, echo=1/1, TR = 2,500.0 ms, TE = 35.0 ms, FA=90.0 deg, SI = 1.00/1.00 mm, FOV = 3.00 cm, MTX = 256, NEX = 4.

Antitumor efficacy in vivo. According to the tumor size, female BALB/c mice with 4T1 breast tumor xenograft were randomly divided into four groups (n=6). The mice were administrated with saline, oxaliplatin, PtFeNP and ETP-PtFeNP (with an equal dose of 5 mg/kg oxaliplatin) at day 14, 17, 20, 23, 26 post implantation. Tumor volume and body weight were recorded every other day. Tumor volume (V_t) was calculated according to the following equation: $V_t = a \times b^2/2$, where a is the longest and b is the shortest axes of the tumor in mm. At the day 28 post implantation, the treated mice were sacrificed. Tumors were isolated from the mice and snap-frozen to -80 ℃ in optimal cutting medium (O.C.T.). The frozen tumor tissue sections were sliced by freezing microtome and mounted on slides. TUNEL assay was performed on the obtained frozen tumor slices by using a One Step TUNEL Apoptosis Assay Kit, following by manufacture' protocol. The stained tumor slices were examined by CLSM.

H&E staining. The main organs (heart, liver, spleen, lung and kidney) of the mice received one-course treatments with different formulations were harvested and fixed with 4 % paraformaldehyde. After routinely paraffin embedding, the organs were sectioned into 10 μm pieces, stained with haematoxylin and eosin, and finally photographed by digital microscopy.

Ototoxicity evaluation. After the fifth injection, the mice treated with different formulations were anaesthetized with chloral hydrate (4%, v/v in saline, 100 μL /20 g body weight) via intraperitoneal injection. All instruments were placed in an anechoic chamber internally covered with acoustic insulating materials. The mice were fixed with a 12-cm distance to the sound source. Three electrodes for detecting neuroelectric pulse were embedded clinging the skull close to auricularis. The audiometric thresholds were recoded according to the pulse change to different sound intensity with variable frequency.

Flow cytometry assay of immune cells population. Tumor draining lymph nodes (TDLN), tumor tissues and spleen were dispersed into single-cell suspensions. Then the TDLN cells, tumor-infiltrating lymphocytes and splenocytes were quantitatively analyzed by flow cytometry following by immunofluorescence staining. Briefly, tissues were harvested and dispersed into single-cell suspensions by using 70 μm cell strainers (BD Pharmingen, New Jersey, America), and then adding 5 mL red blood cell lysis buffer into the suspensions to lysis the red blood cells. After that, cells were collected and dispersed into 200 μL PBS. Before the cells were stained by the addition of a cocktail of fluorescence conjugated antibodies to stain cells for 30 min, 10 μL 1% BSA was added into suspensions to block the non-specific binding. Cells were analyzed via flow cytometric following staining. Fluorescence conjugated antibodies used in flow cytometry are listed in Supplementary Table 1.

IFN-γ detection. Tumor tissues were isolated from mice received one-course treatment with different formulations, homogenated and lysed with RIPA lysis buffer. IFN-γ was analyzed with ELISA kits according to manufacture' protocol.

Statistical Analysis. All the data were presented as means ± standard deviation (SD), and comparison between groups were performed by unpaired t-test. Statistical significance was defined as * $P < 0.05$, ** $P < 0.01$, ***$P < 0.001$.

Reference:

[1] M. Yoshida, J. Mata, J. E. Babensee, J. Biomed. Mater. Res. A **2007**, 80, 7.
